# Viscoelastic Chondroitin Sulfate and Hyaluronic Acid Double-Network Hydrogels with Reversible Cross-Links

**DOI:** 10.1021/acs.biomac.1c01583

**Published:** 2022-02-23

**Authors:** Marko Mihajlovic, Margot Rikkers, Milos Mihajlovic, Martina Viola, Gerke Schuiringa, Blessing C. Ilochonwu, Rosalinde Masereeuw, Lucienne Vonk, Jos Malda, Keita Ito, Tina Vermonden

**Affiliations:** † Department of Pharmaceutics, Utrecht Institute for Pharmaceutical Sciences (UIPS), Utrecht University, 3584 CG Utrecht, The Netherlands; ‡ Department of Biomedical Engineering, Eindhoven University of Technology, 5612 AZ Eindhoven, The Netherlands; § Department of Orthopaedics, University Medical Center Utrecht, Utrecht University, 3508 GA Utrecht, The Netherlands; ∥ Department of Pharmacology, Utrecht Institute for Pharmaceutical Sciences (UIPS), Utrecht University, 3584 CG Utrecht, The Netherlands; ⊥ Department of Equine Sciences, Faculty of Veterinary Medicine, Utrecht University, 3508 GA Utrecht, the Netherlands

## Abstract

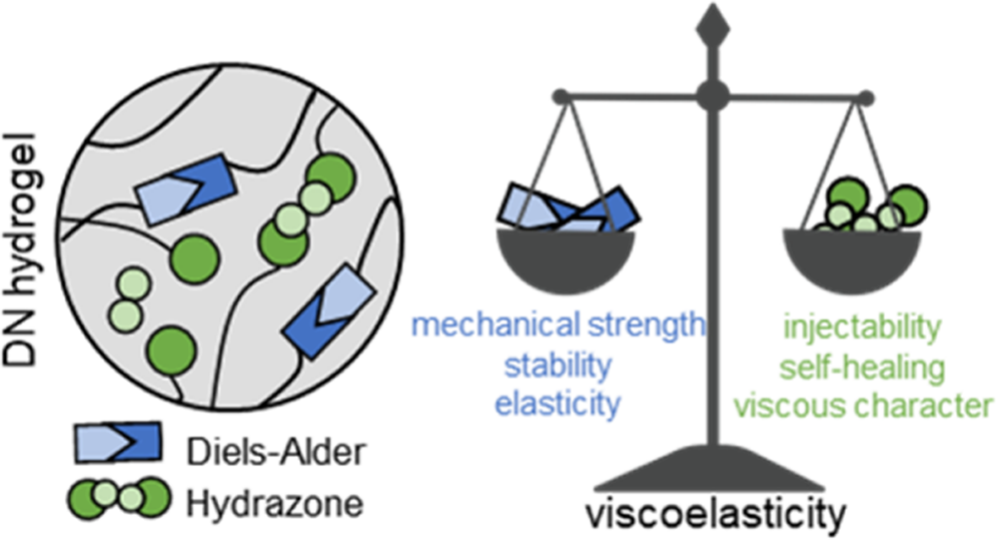

Viscoelastic hydrogels are gaining interest as they possess necessary requirements for bioprinting and injectability. By means of reversible, dynamic covalent bonds, it is possible to achieve features that recapitulate the dynamic character of the extracellular matrix. Dually cross-linked and double-network (DN) hydrogels seem to be ideal for the design of novel biomaterials and bioinks, as a wide range of properties required for mimicking advanced and complex tissues can be achieved. In this study, we investigated the fabrication of chondroitin sulfate/hyaluronic acid (CS/HA)-based DN hydrogels, in which two networks are interpenetrated and cross-linked with the dynamic covalent bonds of very different lifetimes. Namely, Diels–Alder adducts (between methylfuran and maleimide) and hydrazone bonds (between aldehyde and hydrazide) were chosen as cross-links, leading to viscoelastic hydrogels. Furthermore, we show that viscoelasticity and the dynamic character of the resulting hydrogels could be tuned by changing the composition, that is, the ratio between the two types of cross-links. Also, due to a very dynamic nature and short lifetime of hydrazone cross-links (∼800 s), the DN hydrogel is easily processable (e.g., injectable) in the first stages of gelation, allowing the material to be used in extrusion-based 3D printing. The more long-lasting and robust Diels–Alder cross-links are responsible for giving the network enhanced mechanical strength and structural stability. Being highly charged and hydrophilic, the cross-linked CS and HA enable a high swelling capacity (maximum swelling ratio ranging from 6 to 12), which upon confinement results in osmotically stiffened constructs, able to mimic the mechanical properties of cartilage tissue, with the equilibrium moduli ranging from 0.3 to 0.5 MPa. Moreover, the mesenchymal stromal cells were viable in the presence of the hydrogels, and the effect of the degradation products on the macrophages suggests their safe use for further translational applications. The DN hydrogels with dynamic covalent cross-links hold great potential for the development of novel smart and tunable viscoelastic materials to be used as biomaterial inks or bioinks in bioprinting and regenerative medicine.

## 1 Introduction

Soft materials, such as hydrogels (polymeric networks imbibing large amounts of water), hold a huge potential in the design of smart biomaterials, to be used in tissue engineering and regenerative medicine applications, especially in combination with bioprinting techniques. Depending on the polymers used and the specific applications, hydrogels are often found to be biocompatible and biodegradable. As such, hydrogels are good candidates to fabricate scaffolds which are highly hydrated, supporting cellular growth, differentiation, and new tissue formation in vivo.^1−4^ In recent years, the hydrogel design mainly revolved around the use of natural polymers (biopolymers) in order to achieve advantageous interactions between cells and materials. Many biopolymers have been investigated, such as gelatin and different polysaccharides, as they are ubiquitously found in the extracellular matrix (ECM) of many native tissues and are generally bioactive, cytocompatible, and biodegradable. However, to efficiently mimic the complex functions of ECM, hydrogels should possess dynamic (time-dependent), instructive, and responsive features.^5^ Therefore, the hydrogel design should encompass diverse and multiple cross-links, through which functions such as shear-thinning, self-healing, stimuli-responsiveness, and tunable viscoelasticity could be attained. Ideally, the structural design of hydrogels should encompass reversible and dynamic networks rather than covalent, static systems. The choice of a single type of network or cross-linking strategy severely limits the hydrogel in terms of the tunability of properties and functions it can possess. More recently, the use of multiple polymer networks, in addition to carefully chosen cross-linking strategies, emerged as a powerful tool to recapitulate many of the highly desired and sought-after properties. Among the different design approaches of multiple-network-based hydrogels, double-network (DN) hydrogels seem to be promising and an often-used strategy. In DN systems, two networks are interpenetrated, with each network being separately cross-linked.^6,7^ Therefore, there are no additional attachment points between the two networks. An alternative is given by dually cross-linked hydrogels, in which only one network is formed through the combination of multiple cross-linking strategies.^8^ Regarding the design of DN hydrogels, the use of dynamic covalent bonds could be beneficial. Dynamic covalent bonds are considered intermediates between traditional covalent and physical bonds. They possess higher strengths than physical interactions, usually in the order of several 100 kJ/mol, but unlike covalent bonds, they are also reversible and dynamic.^9−11^ Different examples of dynamic covalent bonds as cross-linking functionalities have been used in hydrogels, including Diels–Alder (DA) adducts,^12^ disulfide,^13^ acylhydrazone,^12−16^ oxime,^17^ and boronic ester bonds.^17^

To generate hydrogels in this study, the chosen biopolymers were glycosaminoglycans (GAGs) chondroitin sulfate (CS) and hyaluronic acid (HA), which are linear anionic polysaccharides naturally occurring in human ECM. CS is most abundantly found in connective tissues and in the brain,^18^ whereas HA is most concentrated in synovial fluid and the vitreous of the eye.^18^ Structurally, CS and HA are quite similar, both consisting of repeating disaccharide units, made of d-glucuronic acid (CS and HA) and *N*-acetyl-d-galactosamine (CS) or *N*-acetyl-d-glucosamine (HA). Additionally, CS carries one or more sulfate groups per disaccharide unit, whereas HA is a nonsulfated GAG.^18^ Both of these polymers have been employed to fabricate hydrogels,^19−24^ but HA has seen far more use when compared to CS, and to the best of our knowledge, CS has not been employed in hydrogel formulations based on dynamic covalent bonds and designed for bioprinting applications.^25^ Besides, both CS and HA are found in cartilage tissue, with CS displaying higher water retention capacity than HA,^26^ thus potentially leading to hydrogel scaffolds with significant swelling capacity. Such feature is, for instance, important in mimicking the load-bearing behavior of cartilage tissue, making CS a suitable polymer in biomaterials for cartilage tissue engineering applications. Therefore, in the present work, we mainly focus on the CS polymer, and HA is used to a lesser extent.

Recently, Yu et al. developed an HA/PEG hydrogel system containing both acylhydrazone bonds and DA adducts.^12^ However, strictly speaking, such a system was a dually cross-linked network and not a DN hydrogel (two interpenetrated, separately cross-linked networks), which may affect the tunability of the material’s response and the formulation process. Moreover, no studies on tunable viscoelasticity nor additive manufacturing applications were explored. Wang et al., on the other hand, developed a DN hydrogel based on HA, cross-linked via acylhydrazone and thiol–ene bonds.^27^ The material showed interesting properties, including photostiffening and photopatterning; however, it did require the use of UV light in order to induce the formation of permanent cross-links.

In this work, we make use of dynamic covalent bonds to cross-link CS and HA into a DN hydrogel, in particular DA adducts and acylhydrazone bonds. The DA cycloaddition generally takes place between an electron-rich diene functional group (e.g., furan) and an electron-poor dienophile (e.g., maleimide); it does not require the use of initiators or catalysts, forms no side products, and occurs in an aqueous environment.^28^ The newly formed cyclohexene adduct is reversible by means of retro-DA reaction at elevated temperatures. Moreover, in this study, furan was replaced with a more electron-rich methylfuran moiety, which has been shown to react faster at physiological pH with maleimide when compared to furan.^29−32^ Also, a previous study suggests that the retro-DA reaction was faster than that when furan was used. With these notions in mind, we purposefully chose methylfuran, as we consider that faster transformation and inverse reaction could be beneficial for our goal of obtaining dynamic and viscoelastic hydrogels. Additionally, hydrazone bonds, derived from the reaction between carbonyls and hydrazine functionalities, are also reversible, and the reaction takes place under physiological conditions, making it suitable for hydrogel fabrication.^33^ We anticipated that by combining two different types of dynamic covalent bonds, we could create a hydrogel that is strong and structurally stable but at the same time dynamic and viscoelastic. The more stable DA adducts (long lifetime) provide the materials with structural integrity and mechanical strength while the highly dynamic acylhydrazone bonds (short lifetime) provide for the dynamic character and processability. Moreover, for the stabilization of the DN, no UV or other external triggers are needed, as DA cross-linking takes place spontaneously under physiological conditions. Our main focus was the study of viscoelasticity of the DN and how the material’s response could be tuned, in addition to exploring the potential applications of such a DN hydrogel.

Specifically, CS was functionalized with maleimide, and a separate batch of CS was oxidized to introduce aldehydes, while HA was modified with methylfuran. In addition, adipic acid dihydrazide (ADH) was used to cross-link the aldehyde containing CS. The fabricated hydrogels were investigated for their viscoelasticity and processability. Moreover, considering that both CS and HA are highly charged at physiological pH, we hypothesized that a DN hydrogel formulation based on CS and HA would result in materials with significant swelling capacity. Therefore, one of the interests was also to assess the hydrogel swelling capacity and related mechanical properties, for potential cartilage-mimicking applications. Finally, cytocompatibility was evaluated. The unique assortment of features displayed by these hydrogels, alongside the possibility to tune and customize some of them by varying the composition, makes these gels suitable candidates to mimic complex soft tissues.

## 2 Experimental Section

### 2.1 Materials

Sodium hyaluronate was obtained from Lifecore Biomedical (82 kDa as measured with GPC, Chaska, MN). Chondroitin 4-sulfate sodium salt (bovine trachea, lyophilized powder suitable for cell culture, molecular weight 19.9 kDa as measured previously^34^) was purchased from Sigma-Aldrich (Zwijndrecht, the Netherlands). All other reagents were obtained from either Sigma-Aldrich (Zwijndrecht, the Netherlands) or TCI Europe N.V. and were used as received. All organic solvents were purchased from Biosolve (Valkenswaard, the Netherlands) and used without further purification.

### 2.2 Synthesis

#### 2.2.1 Chondroitin Sulfate-Maleimide (CS-mal) Functionalization

Chondroitin sulfate was functionalized with maleimide moieties by adapting a method previously reported for hyaluronic acid.^35^ CS sodium salt was first converted into CS tetrabutylammonium (TBA) salt, through resin exchange (Dowex 50 × 8w hydrogen form and *tert*-butyl-ammonium fluoride). CS-TBA was obtained after freeze-drying. The molar ratio of TBA ions per disaccharide unit of CS was ∼1.7. Dry CS-TBA (6.0 g, 7.60 mmol disaccharide units) was then transferred in a three-neck round-bottom flask and flushed under N_2_ flow. Next, 1-(2-aminoethyl) maleimide (1.035 g, 5.86 mmol) and benzotriazol-1-yloxytris(dimethylamino)phosphonium hexafluorophosphate (BOP) (3.20 g, 7.23 mmol) were combined with CS-TBA, and anhydrous DMSO (300 mL) was added. The reaction mixture was stirred for 4 h at room temperature. The solution was dialyzed extensively (cutoff 14 kDa), first against NaCl (150 mM) for 4 days and then against water for 3 days. Finally, the solution was lyophilized, and the product CS-mal was recovered as a white fluffy solid (yield ∼90%). The degree of functionalization, defined as the number of ethyl maleimide groups per disaccharide unit (expressed as percentage), was determined by ^1^H NMR.

#### 2.2.2 Hyaluronic Acid-Methylfuran (HA-MeFU) Functionalization

Hyaluronic acid was functionalized with 5-methylfurfurylamine, following a previously reported method.^36^ Briefly, HA was weighed (3.00 g, 7.47 mmol disaccharide units), transferred in a round-bottom flask, and dissolved in MES buffer (100 mM, pH 5.5) at a concentration of 1 w/v % (300 mL). Next, 4-(4,6-dimethoxy-1,3,5-triazin-2-yl)-4-methyl-morpholinium chloride (DMTMM) was added (4.15 g, 14.98 mmol), followed by a dropwise addition of 5-methylfurfurylamine (1000 μL, 8.97 mmol). The reaction mixture was stirred at room temperature for 18 h, dialyzed against NaCl (150 mM) for 2 days and then against water for another 2 days (cutoff 14 kDa). Finally, HA-MeFU was obtained after freeze-drying as a light-yellow, cotton-like solid (yield ∼80%). The degree of functionalization, defined as the number of 5-methylfuran groups per disaccharide unit (expressed as percentage), was determined by ^1^H NMR. The molecular weight of 82 kDa was chosen to allow for the high reproducibility of the functionalization reaction, as well as to ensure not too high viscosity for the following formulation and hydrogel fabrication steps.

#### 2.2.3 Chondroitin Sulfate Oxidation

Chondroitin sulfate was oxidized following a protocol previously published.^37^ Briefly, CS sodium salt (7.0 g, 16.86 mmol disaccharide units) was dissolved in 275 mL of deionized water, followed by the addition of sodium periodate solution (4.2 g, 19.63 mmol, in 75 mL of water). The solution was stirred at room temperature in the dark for 2 h. Subsequently, the reaction was quenched by adding ethylene glycol (14.0 mL, 0.25 mol) and stirring the solution for 1 h. Finally, dialysis against water (cutoff of 14 kDa) for 4 days and freeze drying yielded the final product, oxidized CS (CS-ox), as a white, fluffy solid (yield ∼ 70%).

The degree of oxidation (amount of introduced aldehyde groups) of CS-ox polysaccharide was determined using Purpald reagent, as described previously,^38−40^ with a standard curve from propionaldehyde. Briefly, the CS-ox sample (0.5 mg/mL) and Purpald reagent (10 mg/mL) were dissolved in 1 M NaOH solution. The two solutions were mixed in equal volumes and incubated overnight at room temperature, exposed to air. The absorption of the resulting purple-colored solutions was measured by UV–vis spectroscopy at 550 nm (Shimadzu UV-2450).

### 2.3 Nuclear Magnetic Resonance

^1^H NMR spectra were recorded on an Agilent 400-MR NMR spectrometer (Agilent Technologies, Santa Clara, USA.) in D_2_O. The chemical shifts were reported as δ in parts per million (ppm) and were referenced against the residual solvent peak of D_2_O (δ = 4.79 ppm).

### 2.4 Fourier Transform Infrared Spectroscopy

Fourier transform infrared (FT-IR) spectra were recorded on a PerkinElmer Spectrum Two spectrometer equipped with a universal attenuated total reflectance sampling accessory. The samples were scanned in the range from 400 to 4000 cm^–1^, with eight scans per sample cycle and a resolution of 4 cm^–1^.

### 2.5 Hydrogel Fabrication

Hydrogels were fabricated by dissolving the polymers in phosphate-buffered saline (PBS, 137 mM NaCl, 2.7 mM KCl, 8 mM Na_2_HPO_4_, and 2 mM KH_2_PO_4_, pH 7.4) at desired concentrations (15 wt %, unless otherwise stated) and mixing them together. For single-network (SN)-DA hydrogels, separate solutions in PBS of CS-mal and HA-MeFU were prepared and then thoroughly mixed together. For SN-HY, a solution of CS-ox was made in PBS and then supplemented with ADH (stock solution in PBS, 25 mg/mL). Lastly, DN hydrogel was fabricated by dissolving together CS-mal and CS-ox in PBS, while HA-MeFU was separately dissolved in PBS. Next, the two solutions were combined, mixed thoroughly, and supplemented with ADH to give rise to the DN hydrogel. The molar ratios between the complementary functional groups, as well as mass ratios between DA and HY in DN formulations, are reported in Table S1 and are also indicated throughout the paper. For swelling and mechanical experiments (under free swelling conditions), hydrogel discs were prepared. The combined solutions of dissolved polymers were prepared as described above, followed by injection in a Teflon mold with cylindrical wells (2 mm height and 6 mm diameter), covered with a quartz glass plate, and incubated at 37 °C. Incubation was set at 3 h for SN-DA and DN hydrogels and 5 min for SN-HY. For confined conditions, samples were prepared in the same way, but in addition to using the mold, the wells were fitted with a knitted spacer fabric scaffold (polyamide 6; ∼2.6 mm height, 8 mm diameter, yellow color, and prepared by the warp-knitting method, as previously reported;^41^ KARL MAYER Textilmaschinenfabrik GmbH, Obertshausen, Germany). This results in the formation of the hydrogel within the spacer fabric material (Figure 6A, middle sample). For achieving further confinement, such gel-filled spacer fabric was then placed inside a circular cassette (3 mm height, 8 mm diameter; R05 resin, EnvisionTEC), as shown in Figure 6A (sample on the right). The samples inside the cassette were used for swelling and mechanical tests.

### 2.6 Hydrogel Swelling

Hydrogel swelling properties were assessed by a gravimetric method. The following hydrogel formulations were evaluated over time: SN-DA, SN-HY, and DN (mass ratio between DA and HY of 1:1). Hydrogel discs were prepared at 15 wt % polymer concentration (see above) and placed in preweighed vials. The swelling tests were carried out both under free swelling and confined conditions (as explained in the previous paragraph). The size of the hydrogel samples was 6 mm diameter, 2 mm height for free swelling samples and 8 mm diameter, 2.6 mm height for confined samples. The initial weight (*W*_0_) for all hydrogels was determined immediately after fabrication, and the samples were incubated in 1 mL of PBS (pH 7.4) at 37 °C. At indicated time points, the buffer was removed and the hydrogel weight was measured (*W*_*t*_), followed by replacement with fresh buffer. Swelling ratio (SR, defined as *W*_*t*_/*W*_0_) was used to assess the hydrogel swelling properties over time. All samples/conditions were measured in triplicate.

### 2.7 Rheology

Oscillatory shear measurements were performed using a DHR-2 rheometer (TA Instruments, Etten-Leur, the Netherlands), equipped with a plate–plate (20 mm) measuring geometry and a solvent trap to minimize water evaporation. All measurements were conducted at 37 °C (unless otherwise specified), by pipetting 200 μL of the polymer solution onto the measuring plate. The linear viscoelastic region (LVR) was determined using a strain sweep test at an oscillation frequency of 1 rad/s. Gelation experiments (time sweep) were performed at a frequency of 1 rad/s and a shear strain of 1%. Frequency sweep measurements were conducted at an oscillation strain of 1% (safely within LVR), in the frequency range between 0.01 and 100 rad/s. To evaluate the stress relaxation properties, a step strain of 2% was applied. Dynamic amplitude tests were conducted by alternating the cycles at low strain (1%, 1 min) and high strain (500%, 0.5 min), while keeping the frequency constant at 1 rad/s. To assess shear-thinning, viscosity as a function of shear rate was measured in the range between 0.1 and 25 s^–1^.

### 2.8 Mechanical Tests

To study the mechanical properties of the constructs, an indentation test was performed using a tensile tester (MTS Criterion, Eden Prairie, USA) equipped with a loadcell of 50 N (MTS Systems Corp., Eden Prairie, USA). The samples were placed in a custom-made 3D-printed cassette (resin R05, EnvisionTEC) with a diameter of 8 mm and indented with a 5 mm diameter indenter. After a preload of 2.5 kPa was reached, a stress relaxation test was performed using 15% strain (strain rate of 15% strain per second). The strain was kept constant up to 180 s, until an equilibrium was reached. The initial modulus was calculated from the linear part of the loading curve, between 10 and 15% strain. The equilibrium modulus was determined after the sample reached equilibrium at 15% strain.

### 2.9 3D Printing and Imaging

The DN hydrogel (DA/HY 2:1, 15 wt %) was tested for printing. CS-mal and CS-ox polymers were dissolved together in PBS, separately from HA-MeFU. The polymer solutions were then combined, thoroughly mixed, and transferred in a syringe. Such solution was then supplemented with the ADH cross-linker, and upon the formation of the hydrazone cross-links (within minutes), the formulation was printed with a 27 G needle. Extrusion-based printing was performed using a pneumatic 3D bioprinter (Allevi 3, 3D Systems), equipped with Allevi Bioprint Online software. The GCODE file was exported from the Allevi3D Web site. Parameters such as layer patterns, infills, printing speeds, and pressure were tuned by the software. The constructs (square mesh geometry) were printed in standard Petri dishes (Thermo Fisher Scientific). To obtain good resolution of the fibers, the constructs were printed with a 2 mm infill, printing speed of 5 mm/s, and a pressure of 10 psi. Before the printing process, both the collector plate and the extruder were heated to 37 °C, and the temperature was kept constant throughout the experiment. The constructs were analyzed with an Olympus SZ61 stereomicroscope (Yourtech) using the CellSens software.

### 2.10 Preparation of Hydrogel Degradation Products

Degraded hydrogel products were used to assess its proinflammatory potential. In order to fully degrade the material, DN hydrogel was first prepared, as already described (15 wt %, DA-to-HY mass ratio 1:1). After the DN gel was fully cross-linked, it was placed in excess Milli-Q water and supplemented with hyaluronidase type II enzyme (HYAL II, from sheep testes, Sigma-Aldrich). HYAL II was used at 100 U (where 1 U is the amount of enzyme needed to free 1.0 μmol of *N*-acetylglucosamine from the polymer backbone per minute at 37 °C and at pH 4.0^42^). The HYAL II enzyme is not specific, and it degrades both HA and CS polymers.^43^ The samples were incubated at 37 °C under continuous agitation. Following the hydrogel’s complete dissolution, the resulting solution was freeze-dried to yield a solid, white powder, corresponding to the hydrogel degradation products (HDPs). The formed material was used in further experiments.

### 2.11 Human Mesenchymal Stromal Cell Isolation, Expansion, and Treatments

Mesenchymal stromal cells (MSCs) were derived from bone marrow aspirates from healthy donors (*n* = 3, age range 2–12), as approved by the Dutch Central Committee on Research Involving Human Subjects (CCMO, Bio-banking bone marrow for MSC expansion, NL41015.041.12). The parent or legal guardian of the donor signed the informed consent approved by the CCMO. In brief, the mononuclear fraction was separated using a density gradient (Lympoprep, Axis Shield). The MSCs were isolated by plastic adherence and expanded for three passages in Minimum Essential Media (αMEM, Macopharma) with 5% (v/v) human platelet lysate and 3.3 IU/mL heparin and cryopreserved. Subsequently, the MSCs were expanded for two additional passages in αMEM (Gibco), supplemented with 10% (v/v) fetal calf serum (FCS; Biowest), 100 U/mL and 100 μg/mL penicillin/streptomycin (Gibco), 200 μM l-ascorbic acid 2-phosphate (Sigma-Aldrich), and 1 ng/mL basic fibroblast growth factor (PeproTech). Subconfluent MSC monolayers were treated with individual components or cross-linkers diluted into the medium for 24 h (CS-mal and HA-MeFU at 37.5 mg/mL, CS-ox at 75 mg/mL, and ADH at 1.4 mg/mL). The subconfluent MSC monolayers were also treated with cross-linked DN hydrogels in hanging cell culture inserts (pore size 0.4 μm; Merck). Analyses were performed after 1 and 7 days of culture.

### 2.12 THP-1 Cell Culture, Macrophage Differentiation, and Treatment with HDPs

Human THP-1 monocytes were grown in suspension in complete culture medium consisting of Roswell Park Memorial Institute (RPMI) 1640 medium (Lonza, Verviers, Belgium) supplemented with 10% FCS (Greiner Bio-One, Alphen aan den Rijn, the Netherlands) and 1% penicillin/streptomycin (complete culture medium), at 37 °C and 5% (v/v) CO_2_. Cells were used at passage numbers 8–15. The cells were subcultured twice a week in order to maintain a density of approximately 0.5 × 10^6^ cells/mL and were seeded at a density of 1.5 × 10^5^ cells/cm^2^ prior to macrophage differentiation. Macrophage differentiation was achieved by exposing the cells to phorbol 12-myristate 13-acetate (PMA; 50 ng/mL; InvivoGen, the Netherlands) for 48 h (M0 macrophages), and polarization to M1 macrophages was reached after a resting phase of 48 h in complete culture medium and subsequent exposure to lipopolysaccharide (LPS from *Escherichia coli* 0127:B8 100 ng/mL) and interferon gamma (IFNγ; 20 ng/mL; InvivoGen, the Netherlands) for 72 h. The cell medium was changed prior to and after each exposure. Following the macrophage differentiation and polarization process, M0 and M1 macrophages were exposed for 24 h to a range of concentrations of HDPs dissolved in complete culture medium and filter-sterilized.

### 2.13 Cell Viability Assays

#### 2.13.1 THP-1

Following 24 h exposure to the increasing concentrations of HDPs (0.001–15 mg/mL) in 96-well plates, cytotoxicity was measured using CyQUANT LDH cytotoxicity assay (Invitrogen, Thermo Fisher Scientific) according to the manufacturer’s instructions. Triton X-100 (1%) was used as a positive control, and complete culture medium was used as a blank. The absorbance at 490 nm was measured using a GloMax Discover microplate reader (Promega, Madison, WI, USA). Data were corrected for the background (blank) and presented as average absorbance values at 490 nm.

#### 2.13.2 MSCs

MSCs from three donors (two males and one female, age range 2–12, passage number 5) were preseeded 1 day before the start of the experiment in 24-well plate wells at 20,000 cells per well in MSC expansion medium. Upon the start of the experiment, the medium was changed for fresh expansion medium. Cell viability following 1 and 24 h treatment with individual gel components and 1 and 7 days’ treatment with DN hydrogels was assessed using the Live/Dead Viability Kit for mammalian cells (Thermo Fisher Scientific) according to the manufacturer’s instructions. The monolayers were imaged using an Olympus IX53 fluorescent microscope, and live and dead cells were quantified using ImageJ software version 1.49 (National Institutes of Health, Bethesda, MD, USA). In addition, MSCs exposed to DN hydrogels were assessed for metabolic activity and lactate dehydrogenase (LDH) release. The metabolic activity was evaluated using a resazurin (Alfa Aesar, Germany) assay at 44 mM in the culture medium. The fluorescence of reduced resorufin was measured after 6 h of incubation at 544 nm excitation and 570 nm emission using a spectrofluorometer (Fluroskan Ascent FL; Thermo Fisher) and corrected for blanks. The activity of LDH released into the culture medium was measured using the Cytotoxicity Detection KitPLUS (Roche) according to the manufacturer’s instructions. Control wells were treated with 2% Trition X-100. Monolayers were lysed by three freeze–thaw cycles in Tris–EDTA buffer (10 mM Tris–HCl, 1 mM EDTA, pH 8), and DNA was quantified using the Quant-iT PicoGreen assay (Invitrogen) according to the manufacturer’s instructions.

### 2.14 Enzyme-Linked Immunosorbent Assay

The production of interleukin 6 (IL-6), tumor necrosis factor alpha (TNF-α), interleukin 1 beta (IL-1β), and monocyte chemoattractant protein 1 (MCP-1) by macrophages was measured using enzyme-linked immunosorbent assay (ELISA). Cell culture supernatants were collected after 24 h treatments of M0 and M1 THP-1-derived macrophages with HDPs (0.005–0.5–5 mg/mL) and centrifuged for 10 min, 240*g*, 4 °C, and stored at −20 °C until further measurements. DuoSet ELISA Development Systems kit (#DY201; R&D Systems, Abingdon, UK) was used to quantify the IL-1β levels in complete cell culture medium supernatants following manufacturer’s instructions. Standard TMB ELISA Development kits were used to quantify the remaining cytokines (IL-6 #900-T16, TNF-α #900-T25, and MCP-1 #900-T31; PeproTech, London, UK) according to the manufacturer’s protocols. The optical density was determined using a GloMax Discover Microplate Reader (Promega, Madison, WI, USA) set to 450 nm. Each sample was measured in duplicate, and quantification was done using a four-parameter logistic (4-PL) curve fit. Data were presented as concentration (pg/mL) normalized to DNA content quantified using Quant-iT PicoGreen assay, as described above.

### 2.15 Statistical Analysis

All data are presented as mean ± standard error of the mean (SEM). Statistical analysis was performed using one-way ANOVA, followed by either Dunnett’s or Tukey’s multiple comparison tests. A *p*-value < 0.05 was considered significant. The software used for statistical analysis was GraphPad Prism (version 8.43; GraphPad software, La Jolla, CA, USA).

## 3 Results and Discussion

### 3.1 Synthesis and Characterization of HA and CS Derivatives

**Figure 1:**
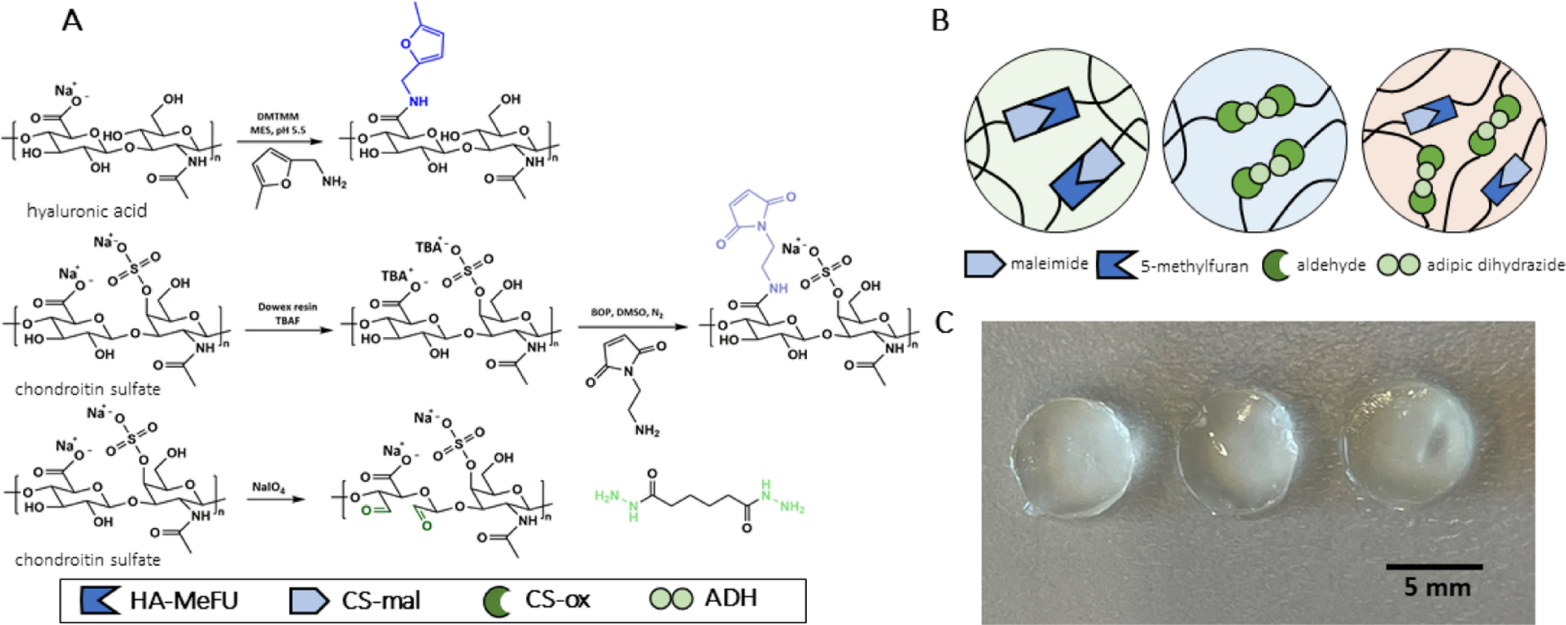
Modification of CS and HA polymers and hydrogel formulations. (A) Synthesis routes for the preparation of HA-MeFU, CS-mal, and CS-ox, including the structure of ADH. (B) Schematic representation of the polymer networks of the hydrogels; SN-DA through coupling between the maleimide and methylfuran functionalities; SN-HY (SN hydrazone) formed in a reaction between the aldehydes of CS-ox and hydrazides of ADH; DN hydrogel formed when all components are present, containing both the DA and hydrazone cross-links. (C) Hydrogels upon in situ cross-linking at 37 °C; from left to right: SN-DA (15 wt %, methylfuran-to-maleimide ratio of 5:1), SN-HY (15 wt %, aldehyde-to-hydrazide molar ratio of 1:1), and DN hydrogels (15 wt %, SN-DA-to-SN-HY mass ratio of 1:1, methylfuran-to-maleimide of 5:1, and aldehyde-to-hydrazide of 1:1), prepared in a Teflon mold (6 mm diameter and 2 mm height).

For the formation of DA cross-links, the 5-methylfuran and maleimide moieties are introduced on HA and CS polymers, respectively. The HA furan derivative (HA-MeFU) was prepared by means of a simple, one-step reaction between HA polysaccharide and 5-methylfurfurylamine, in the presence of 4-(4,6-dimethoxy-1,3,5-triazin-2-yl)-4-methylmorpholinium chloride (DMTMM) reagent (Figure 1A).^36^ The degree of modification was determined by ^1^H NMR, and it was found to be ∼30% (30 in 100 disaccharide repeating units are functionalized with furan), and all signals are in line with the proposed structure (Figure S1). Moreover, successful modification with furan was confirmed by FT-IR measurements (Figure S2A). In fact, upon the conjugation of 5-methylfurfurylamine to the carboxylate groups of HA, the signal corresponding to the carbonyl stretch was shifted from 1617 to 1652 cm^–1^, confirming the formation of amide bonds. A new signal at 795 cm^–1^, corresponding to alkene bending, also indicates the presence of furan.^36^

The CS maleimide derivative (CS-mal) was prepared following a two-step approach. First, CS sodium salt was converted to a more lipophilic salt (CS-TBA) through a resin exchange step to enable the dissolution of CS in DMSO. The next step was the coupling reaction between 1-(2-aminoethyl) maleimide and the carboxylate of CS, in the presence of BOP as the coupling reagent (Figure 1A). The successful formation of CS-mal was confirmed by ^1^H NMR (signals in line with the proposed structure) and FT-IR analyses. The degree of modification was measured to be ∼8% (Figure S1). The FT-IR spectra showed a signal at 1704 cm^–1^ for the carbonyl stretch, and at 695 cm^–1^, the presence of alkene bending (indicative of maleimide) was observed.^36^ Although maleimide-functionalized HA has been reported earlier, to the best of our knowledge, this is the first time direct coupling of maleimide groups to CS is described.^35^

For the formation of the second type of cross-links, hydrazone bonds were employed. Consequently, aldehyde groups were introduced on CS following a previously reported method,^37^ using NaIO_4_, which led to ring opening of glucuronic acid and introduction of dialdehyde functionalities (Figure 1A). Aldehyde formation was confirmed by FT-IR spectra, where a shoulder peak at 1731 cm^–1^ was observed (corresponding to the aldehyde carbonyl stretch)^44,45^ (Figure S3). Quantification of the aldehyde groups was carried out by the Purpald method, confirming an oxidation degree of ∼10% (10% of disaccharide units contain aldehyde moieties). This value is in line with the previously reported results for the oxidation of CS and HA polymers when using a 1.2:1 M ratio of NaIO_4_ to disaccharide unit.^37,44^ The oxidation procedure has been reported to affect the molecular weight of CS^37^ and alginate,^46^ leading to chain fragments below 3.5 kDa in size. In the present study, it is evident that when 1.2 equiv of NaIO_4_ was used, no excessive chain cleavage took place, as 70% of the polymer was recovered after dialysis (MWCO 14 kDa). In order to form a hydrogel with CS-ox, hydrazone cross-links are formed between aldehydes and hydrazide functionalities. In the present work, a bifunctional hydrazide, adipic acid dihydrazide (ADH), was used in combination with CS-ox (Figure 1A). Overall, ^1^H NMR and FT-IR analyses confirmed the successful modification of the HA and CS polysaccharides.

The following nomenclature is used in this work to distinguish the hydrogel samples by their composition: SN-DA for the SN hydrogel containing only DA cross-links (between the methylfuran and maleimide groups of HA-MeFU and CS-mal, respectively), SN-HY for the SN hydrogel cross-linked only by hydrazone bonds (between the aldehyde and hydrazide functionalities of CS-ox and ADH), and finally DN for the DN hydrogel containing both types of cross-links (DA and hydrazones), meaning DN is a four-component system. A schematic representation of hydrogel-constituting networks, made by different types of cross-links, is shown in Figure 1B. Figure 1C shows a representative picture of the SN-DA, SN-HY, and DN hydrogels formed in situ at 37 °C. The HA and CS polymers of similar molecular weights and hydrophilicity were chosen to ensure that no phase separation takes place, as demonstrated by the nearly transparent nature of the hydrogels.

### 3.2 SN Hydrogel Formation and Rheological Characterization

**Figure 2:**
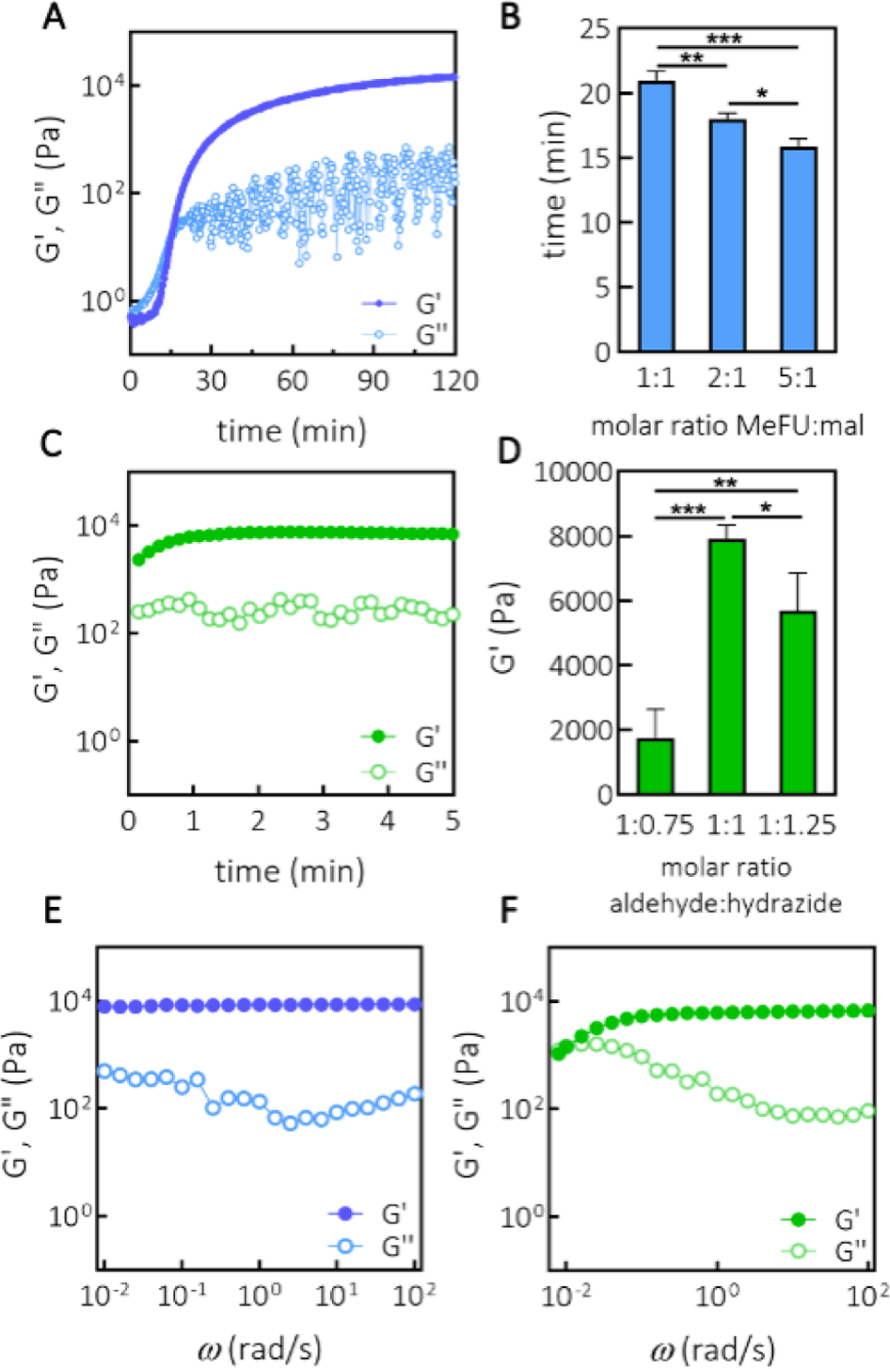
Formation of SN hydrogels and rheological characterization. (A) Time sweep (ω = 1 rad/s, γ = 1%) of SN-DA at 15 wt % (methylfuran-to-maleimide ratio of 5:1). (B) Relation between gelation time (cross-over point) and molar ratio between methylfuran and maleimide, as determined from the time sweep measurements, *n* = 3. (C) Time sweep (ω = 1 rad/s, γ = 1%) of SN-HY at 15 wt % (aldehyde-to-hydrazide molar ratio of 1:1). (D) Storage modulus *G*′(ω) of SN-HY as a function of molar ratio between aldehyde and hydrazide moieties, as determined from the time sweep measurements, *n* = 3. (E) Frequency sweep (ω = 0.01–100 rad/s, γ = 1%) of SN-DA at 15 wt % (methylfuran-to-maleimide ratio of 5:1). (F) Frequency sweep (ω = 0.01–100 rad/s, γ = 1%) of SN-HY at 15 wt % (aldehyde-to-hydrazide molar ratio of 1:1). *p < 0.05, **p < 0.01, ***p < 0.001 (One-way ANOVA, Tukey’s multiple comparison test).

Using shear oscillatory rheometry, the gelation kinetics of SN-based hydrogels were investigated. Specifically, SN-DA hydrogels prepared with 15 wt % polymer content (5:1 molar ratio of methylfuran to maleimide) displayed a cross-over point between *G*′(ω) and *G*″(ω) at ∼15 min, indicating the progress of the cross-linking process and formation of the DA adducts between the methylfuran and maleimide groups. The storage modulus reached a plateau value of ∼15 kPa after 120 min (Figure 2A). The effect of the ratio between methylfuran and maleimide functionalities on the kinetics of gelation [gelation time, here defined as the time needed to reach the cross-over point (*G*′ > *G*″)] was further evaluated. By increasing the ratio of methylfuran to maleimide from 1:1 to 2:1 and 5:1, a decrease in gelation time was observed. Specifically, the ratio of 1:1 required ∼21 min to gelate, whereas for 2:1 and 5:1 ratios, the gelling time was ∼18 and 15 min, respectively (Figure 2B). These enhanced gelation kinetics suggest that an excess of methlyfuran moieties leads to a more rapid reaction with the maleimide groups and thus more rapid formation of the DA cross-links. Using the ratios with excess maleimide to furan would have most likely led to similar trends in gelation kinetics; however, in the current study, these were not investigated as an excess of maleimides could lead to unwanted side reactions with biological components. Unlike maleimide, the excess of furan moieties is not expected to have a negative impact on the application in biological fluids.

Moreover, the gelation of the SN-HY hydrogel was also assessed. A hydrogel was formed instantly upon the addition of an ADH cross-linker to the solution of CS-ox (1:1 aldehyde to hydrazide, 15 wt % CS-ox) (Figure 2C). These kinetics are faster than that in the study of Hafeez et al., where oxidized alginates took ∼45 min to form hydrazone cross-links, at a polymer concentration of 2% (*w*/*v*), although the difference in polymer concentrations likely plays a role in the kinetics as well.^47^ Further, by changing the ratio between the aldehyde and hydrazide functionalities to 1:0.75 and 1:1.25, hydrogels were also instantly formed; however, they showed a lower storage modulus. No gelation was observed upon deviating further in either direction from this range of ratios. Specifically, we found that the optimal ratio of aldehyde to hydrazide was 1:1, leading to the stiffest gels (∼8000 Pa, at 15 wt %) (Figure 2D). Although, hemiacetal formation between aldehydes and hydroxyl functionalities has been reported in polysaccharides,^48^ it is not likely to be significant in this system, as no network formation was observed, unless ADH was added to the CS-ox solution.

The general viscoelastic behavior of the hydrogels was examined by performing a frequency sweep measurement on the SN-DA and SN-HY hydrogels. For SN-DA, it is quite apparent that in the frequency range examined (ω = 0.01–100 rad/s), there was frequency independence of *G*′(ω), whereas *G*″(ω) showed a very weak dependence in the same frequency range, displaying a mild upturn at lower frequencies (Figure 2E). Such a pattern is very typical of and often observed in chemically cross-linked hydrogels, which is expected, considering that the DA reaction leads to covalent bond formation.^49^ A similar behavior was observed with other hydrogels based on DA cross-linking.^50^ In contrast, SN-HY shows a completely different behavior. Namely, in the same frequency range, SN-HY exhibited a significant variation of moduli (Figure 2F). At lower frequencies, *G*″(ω) showed a significant upturn, while *G*′(ω) started decreasing, indicating structural relaxation processes in the network, eventually leading to a cross-over point between *G*′(ω) and *G*″(ω). This is an indication of a dynamic and nonpermanent nature of the SN-HY network, which is in line with the reversible character of the hydrazone bonds.^33^ The cross-over point is observed at ω = 8.4 × 10^–3^ rad/s, and from this, a characteristic relaxation time for the hydrazone network τ = 750 s is derived (τ = 1/*f*, where *f* is frequency).

Strain sweep measurements (γ = 0.01–1000%) were carried out for both SN-DA (methylfuran-to-maleimide of 5:1, 15 wt %) and SN-HY (aldehyde-to-hydrazide of 1:1, 15 wt %) hydrogels to determine the extension of the LVR for these materials (Figure S4). The results showed that yielding strains of ∼50 and ∼80% were observed for SN-DA and SN-HY, respectively, confirming that all oscillatory shear experiments were performed at strain values safely within the LVR.

To further test the reversible character of the hydrazone cross-links, the SN-HY hydrogel was subjected to a dynamic amplitude test (Figure S5). In an alternating fashion, low (γ = 1%) and high (γ = 500%) shearing were applied, and the moduli were monitored in time. Initially, at low strain, the hydrogel exhibited a gel-like structure, with both *G*′(ω) and *G*″(ω) being stable in time. Next, by applying strain values well outside the LVR, *G*″(ω) became higher than *G*′(ω), suggesting a lack of gel structure, due to the breaking of hydrazone cross-links under shear. Finally, by restoring the strain back to the linear region, there was an immediate recovery of *G*′(ω) and the gel structure, indicating that hydrazone bonds were reformed. Clearly, the SN-HY hydrogel exhibits yield behavior that is reversible, making the gel also self-healing.^51^

Based on the gelation results, 5:1 methylfuran-to-maleimide and 1:1 aldehyde-to-hydrazide ratios were chosen for further investigation of the DN hydrogels.

### 3.3 DN Hydrogel Formation and Rheological Characterization

**Figure 3:**
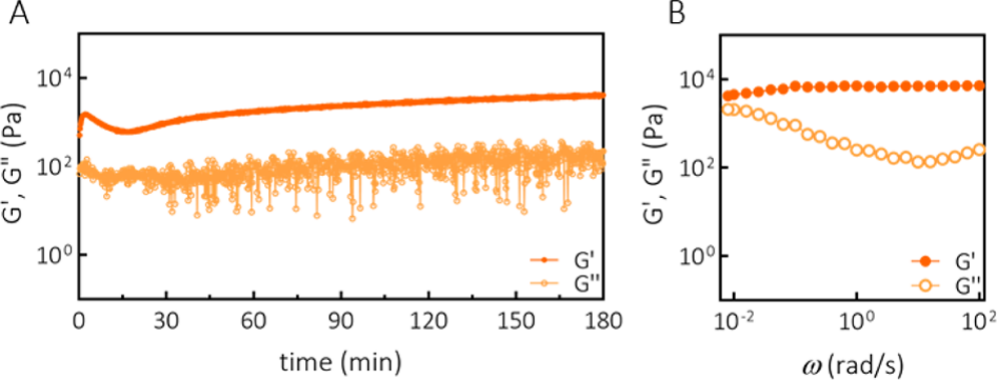
Formation of DN hydrogel and rheological characterization. (A) Gelation (ω = 1 rad/s, γ = 1%) of DN at 15 wt % (SN-DA-to-SN-HY mass ratio of 1:1, methylfuran-to-maleimide molar ratio of 5:1, and aldehyde-to-hydrazide molar ratio of 1:1). (B) Frequency sweep (ω = 0.01–100 rad/s, γ = 1%) of DN at 15 wt % (SN-DA-to-SN-HY mass ratio of 1:1, methylfuran-to-maleimide molar ratio of 5:1, and aldehyde-to-hydrazide molar ratio of 1:1).

Following the optimization of gelation of the SN hydrogel formulations (SN-DA and SN-HY), DN hydrogels were prepared and characterized. A mass ratio between the two networks was set at 1:1 at 15 wt % polymer content (keeping the molar ratio between the functional groups as previously optimized; methylfuran to maleimide, 5:1 and aldehyde to hydrazide, 1:1), and the gelation was monitored in time. Figure 3A shows the gelation profile for the DN hydrogel. It is evident that immediately after the start of the measurement, SN-HY was being formed, as *G*′(ω) was higher than *G*″(ω). Following the formation of hydrazone cross-links, the network started relaxing, likely due to a very fast exchange of hydrazone bonds between bound and unbound states. Such a decrease in moduli could be related to the hydrolysis of the polysaccharide and thus the depletion of available aldehydes. It has been reported that the ring-opened structures of polysaccharides (such as di-aldehyde structures obtained by periodate oxidation) are susceptible to hydrolysis.^44,52,53^Figure S6A shows the gelation of SN-HY (7.5 wt %, aldehyde-to-hydrazide ratio of 1:1) with also a noticeable decrease of moduli after the quick gelation. Next, after ∼15 min, the moduli started increasing further, suggesting that the DA cross-links were formed in sufficient amounts to affect the material’s stiffness as seen with SN-DA (Figure 2A). The gelation followed slower kinetics compared to that of SN-DA, which is most likely related to a lower fraction of HAMeFU and CS-mal polymers (thus methylfuran and maleimide functionalities) in the DN formulation. After 2–3 h, the DN seemed to be fully cross-linked and equilibrated, showing a final modulus of ∼6 kPa. *G*′(ω) is directly related to the cross-linking density of the network; therefore, a lower modulus found for DN compared to SN-DA can be attributed to the lower density of DA cross-links in DN (as the concentration of the reactive groups was halved compared to the SN-DA formulation). After 3 h, the hydrazone cross-links were not expected to contribute significantly to the overall cross-linking density and stiffness of the DN, as within 6 min, the moduli already started to decrease (Figure S6A). The results in Figure S6B show the extension of the LVR for the DN hydrogel, with a yielding strain of ∼60%.

Furthermore, being very dynamic, hydrazone bonds could be used to allow for material processing before the dynamic covalent cross-linking of DA becomes dominant. In order to test whether the final hydrogel properties and gelation process are affected by shearing of the first-formed HY cross-links, a gelation experiment with a short initial shearing was performed. This simulates the gelation process of DN in case the initially formed SN-HY hydrogel is destroyed before the DA reaction is complete.

Figure S6C shows the gelation of the DN with initial shearing applied. This shearing resulted in the temporary destruction of the hydrazone cross-links (*G*″(ω) > *G*′(ω)), but as soon as the shear was removed, the HY cross-links reformed, recovering the gel’s stiffness (Figure S6D). However, the gelation kinetics were not affected, and the final mechanical properties of the DN were not different compared to the gelation without shearing (Figure 3A), suggesting that the hydrogel in its initial gelling stages could be processed, for example, extruded, and still maintain its gelation capacity. A potential benefit of the initial processability of the DN could be employed during the 3D printing processes of this hydrogel formulation. This aspect is further described in the following sections.

Furthermore, the viscoelastic response as a function of frequency of the DN was assessed (Figure 3B). In the frequency range investigated (ω = 0.01–100 rad/s), the loss modulus *G*″(ω) displayed a significant upturn at lower frequencies, indicating a viscous character. This frequency dependence of *G*″(ω) is due to the presence of highly dynamic hydrazone cross-links in the network. On the other hand, the storage modulus *G*′(ω) showed only a weak dependency in the frequency range investigated, indicating a more elastic and more permanent nature of the gel, similar to that observed with SN-DA. Even at the lowest frequency measured (0.01 rad/s), *G*′(ω) was larger than *G*″(ω), in contrast to the SN-HY system. This frequency sweep test suggests that the general viscoelastic behavior of the DN is in between those corresponding to the SNs consisting purely of DA or HY cross-linked hydrogels. Clearly, the DN gel exhibits quite significant viscous properties (due to hydrazone cross-links), while it still maintains the elastic character as well, resulting in a stable structure in time (due to DA cross-links).

### 3.4 Tunable Stress Relaxation and Viscoelastic Properties

**Figure 4:**
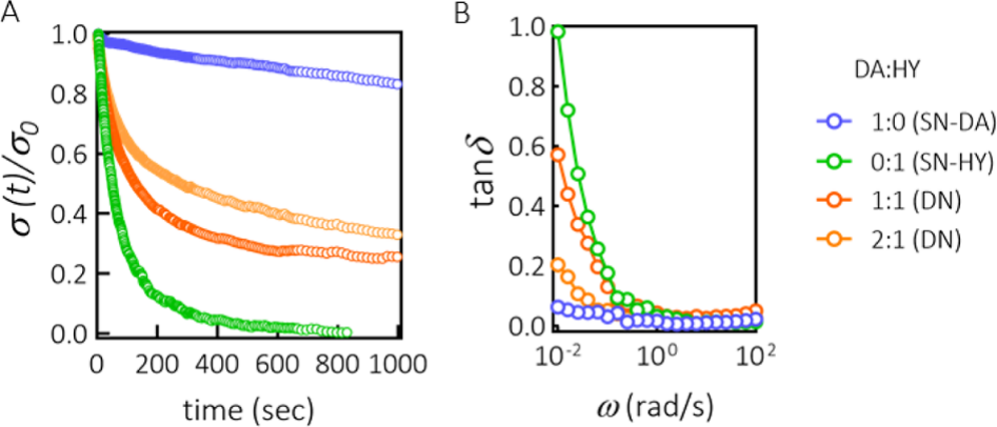
Tunable viscoelastic properties of DN hydrogels. (A) Stress relaxation of different hydrogel formulations after a step strain of γ = 2% (measurements performed on fully cross-linked hydrogels). All hydrogel formulations were prepared at 15 wt % polymer concentration (the mass ratio between DA and HY was varied as indicated, whereas the molar ratios of methylfuran to maleimide and aldehyde to hydrazide were kept at 5:1 and 1:1, respectively). (B) Loss factor tan δ, defined as the ratio *G*″(ω)/*G*′(ω), as a function of angular frequency (calculated from the corresponding frequency sweep measurements).

Stress relaxation is an important feature of viscoelastic materials to investigate as they display time-dependent mechanical response. Figure 4A shows the stress relaxation response of both SN and two formulations of DN hydrogels (DA/HY ratios of 1:1 and 2:1), measured at 37 °C, following a step strain of 2%. In the SN-DA hydrogel, being covalently cross-linked, the stress was dissipated very slowly. Over 16 min, the amount of stress dissipated was ∼17%. For the chemically cross-linked hydrogels, the stress relaxation is likely due to the changes in the conformation of chains in the network. In contrast to SN-DA, both SN-HY and DN hydrogels showed a two-stage response. At short times, there was a quick stress relaxation, which is related to the conformational changes of chains, but in addition to that, there was also a structural reorganization of the network due to the reversible nature of the hydrazone cross-links. Dissociation of the hydrazone cross-links in the network led to efficient and fast dissipation of elastic energy, resulting in 50% of stress released after only ∼45 s for SN-HY, whereas for DN 1:1 and DN 2:1, it took ∼115 and 250 s, respectively (Figure 4A). The overall trend suggests that it is possible to tune the stress relaxation behavior of the hydrogels depending on the composition, more specifically on the ratio between DA and HY cross-links. Interestingly, for SN-HY, the complete relaxation of the network was achieved after ∼810 s, which is in accordance with the previously discussed characteristic relaxation time from the frequency sweep data (Figure 2F).

Furthermore, the tunable viscoelastic response of hydrogels was evaluated by plotting the loss tangent (tan δ) against frequency (Figure 4B). The observed trend was consistent with the data obtained from the stress relaxation measurements. The upturn of tan δ at low frequencies indicates an increase of viscous contribution to the hydrogel’s response. Specifically, at the lowest frequencies probed, tan δ was 1 for SN-HY (cross-over point), whereas for DN gels, it varied between ∼0.2 and 0.6, depending on the ratio between DA and HY cross-links. In contrast, for SN-DA, the response was predominantly elastic and less frequency-sensitive, with tan δ varying from ∼0.01 to 0.06. Concluding, the lifetime of the DA cross-links is longer than the slowest timescales probed in our experiments, whereas the lifetime of HY cross-links could be determined by the oscillatory shear experiments in this study. SN-DA was not effective to dissipate elastic energy quickly, whereas SN-HY displayed complete and fast relaxation. DN hydrogels were effective in energy dissipation, while still preserving structural stability. The significance of tunability of stress relaxation and mechanical properties of hydrogels by means of dynamic covalent bonds (hydrazone and oxime) has been highlighted in a recent work by Morgan et al.^54^

### 3.5 Injectability and 3D Printing

**Figure 5:**
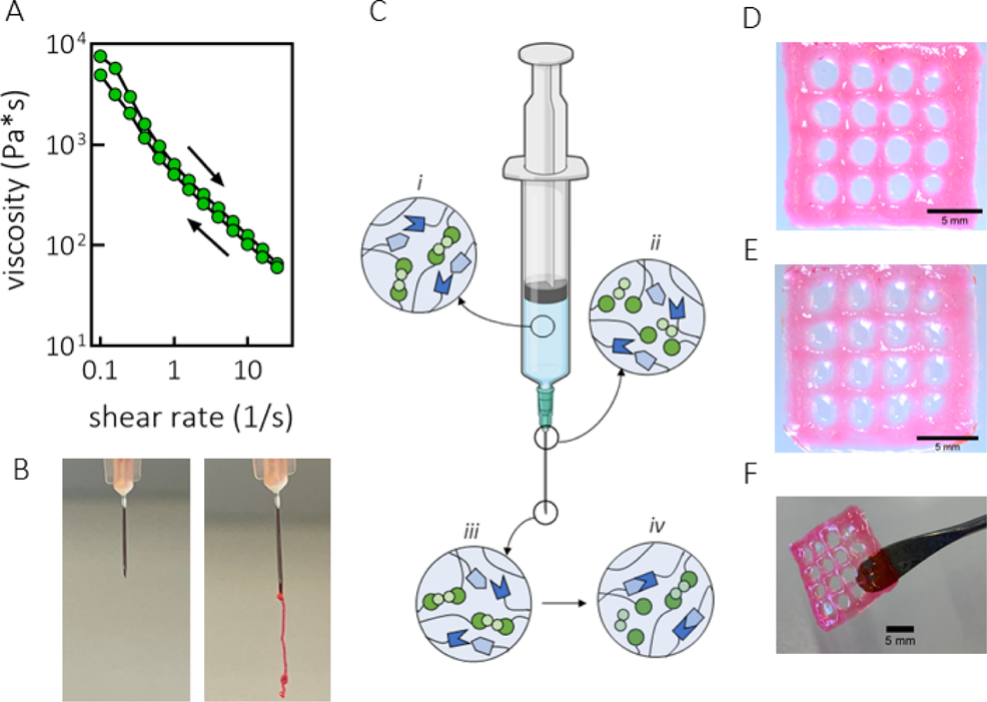
Injectability, flow behavior, and extrusion-based 3D printing. (A) Viscosity as a function of shear rate for the SN-HY hydrogel (15 wt %, aldehyde-to-hydrazide molar ratio of 1:1). Consecutive runs of increasing (0.1–25 1/s) and decreasing shear rates (25–0.1 1/s) were performed. (B) Extrusion of SN-HY hydrogel formulation (10 wt %, aldehyde to hydrazide molar ratio 1:1) through a 27G needle and syringe, confirming the hydrogel’s injectability. Rhodamine dye was added for visualization. (C) Schematic representation of the network structure of DN formulation during the extrusion process; (i) prior to extrusion, in the syringe, HY bonds are formed quickly after the addition of the ADH cross-linker to the formulation, while DA hardly contributes to the network in this stage; (ii) during extrusion through a needle, there is transition from a solid-like to liquid-like state, supported by the dissociation of HY cross-links under shear; (iii) immediately after extrusion, HY cross-links are reformed, going back to the original, solid-like state; (iv) over time, more DA adducts are being formed, leading to a continuous fixation of the hydrogel structure. Created with BioRender.com. (D) Image of a 3D-printed lattice construct of DN hydrogel (15 wt %, DA-to-HY mass ratio of 2:1, methylfuran-to-maleimide and aldehyde-to-hydrazide molar ratios of 5:1 and 1:1, respectively), taken ∼30 min after printing (following DA cross-linking). (E) Image of the same 3D-printed lattice structure of DN taken the following day, after being kept in a humid container overnight and then immersed in PBS for ∼30 min. (F) Self-supporting 3D-printed construct of DN hydrogel displaying structural integrity and stability. Rhodamine dye was added for visualization.

After evaluating the viscoelastic behavior of the SN and DN hydrogels and the dynamic nature of the HY cross-links, we further investigated the extrusion-based processability of hydrogels and consequently the material’s feasibility for 3D printing applications. Generally, for a material to be injectable, the stress applied should be sufficient to induce yielding of the material and consequently its transition from a solid-like to a liquid-like state. Additionally, once the material under stress becomes liquid-like, its viscosity should be low enough to allow for the material to flow when a reasonable force is applied to the piston. Because in the DN hydrogel DA cross-links are not very dynamic under testing conditions and at experimental timescales, injectability and flow behavior of the DN formulation were assessed during the time frame when HY cross-links were formed, but the majority of the DA cross-links were not yet formed. Therefore, we hypothesized that the DN hydrogel could be processed in the first 10–15 min upon mixing of the polymers. Injectability and flow behavior were evaluated first for the SN-HY hydrogel. As observed from the strain sweep experiment for SN-HY (Figure S4B), the hydrogel displayed yielding behavior and thus nonlinear response at strain values above ∼80% with decreasing *G*′(ω) and *G*″(ω). However, the actual transition from solid-like to liquid-like form takes place at higher strain values, when *G*″(ω) > *G*′(ω), which for SN-HY was observed at ∼200% strain (at 1 rad/s). As the material becomes fluidic under shear, its capacity to flow was assessed by measuring the viscosity as a function of the shear rate. As shown in Figure 5A, the viscosity dramatically decreased upon increasing the shear rate from 0.1 to 25 s^–1^ with ∼2 orders of magnitude, indicating shear-thinning behavior. This observation is in agreement with the previously reported hydrazone-cross-linked systems.^27,55^ A consecutive run with decreasing shear rate was also performed, showing that the original viscosity was recovered at low shear rates.^56^

Additionally, we tested whether the SN-HY could be injected through a 27 G needle (Figure 5B). In fact, by applying a gentle force, the hydrogel could be injected. Immediately after leaving the needle, the material appeared solid-like, indicating that the hydrogel structure was recovered (due to the reformation of the HY bonds). Therefore, both the yielding and shear-thinning properties of the SN-HY enabled injectability.

The schematic representation shown in Figure 5C explains in more detail what happens at a molecular level as the DN hydrogel formulation is being processed (e.g., injected). At first instant, after transferring the solution of solubilized polymers (CS-mal, HA-MeFU, and CS-ox), the ADH cross-linker is added. This addition results in the immediate formation of HY cross-links and SN-HY formation, while CS-mal and HA-MeFU react much slower, and the majority of these bonds are not formed yet. At this point, the force is applied and the material can be extruded through a needle. Under shear, the HY cross-links disassemble, and the hydrogel undergoes a transition from the solid to liquid state. Immediately upon exiting the needle, the material recovers to its original, gel-like state due to HY bond reformation, thus enabling the formulation to maintain its gel-like structure until the DA cross-links are formed. After few minutes, DA covalent cross-linking leads to the fixation of the structure. This approach has been employed before for similar systems based on HA polymers, but photopolymerization was required to induce the stabilization of the network.^27^ In addition, Rodell et al. developed a HA-based physical hydrogel based on guest–host interactions, which was further covalently cross-linked via methacrylate-mediated photopolymerization, thus needing UV light.^57^ In the present work, thanks to the spontaneous DA reaction, such fixation and stabilization of the double-cross-linked hydrogel take place in situ, under physiological conditions. SN-HY could also be processed in this way, but the stability over time of the ejected hydrogel was compromised, due to the fast exchange kinetics of HY bonds and the lack of stability offered by the DA cross-links (data not shown).

For initial printing experiments, we used the DN formulation at 15 wt % polymer content, with the DA-to-HY mass ratio of 2:1. Upon the solubilization of polymers, the solution was transferred in the syringe and ADH cross-linker was added, resulting in SN-HY formation within few minutes. At this point, the hydrogel formulation was extruded through a 27 G needle into a four-layer lattice construct with 2 mm spacing between the filaments. Due to the dynamic nature of HY cross-links and their reassociation, the hydrogel maintained its shape and integrity upon ejection (Figure 5D). Next, formation of DA cross-links followed, leading to the stabilization of the structure. However, shape fidelity could get slightly compromised. If ADH is added immediately upon the dissolution of the polymers, SN-HY would be formed immediately, and by the time that DA has progressed enough, the printed construct could start deforming. The printed construct was then kept in a humid container overnight, to allow for DA cross-linking to complete. During that time, the filaments remained stable and did not deform. Subsequently, the hydrogel construct was placed for ∼30 min in PBS. As expected, the hydrogel remained stable due to the DA covalent cross-links, although it did seem to swell a little bit (Figure 5E). Overall, the DN formulation was confirmed to be injectable and processable in its initial stages, whereas upon the Diels–Alder reaction between CS-mal and HA-MeFU, the structure was rendered robust. The printed construct was shown to maintain shape fidelity as well as structural integrity, and it was able to support its own weight (Figure 5F).

### 3.6 Swelling Behavior, Stability, and Mechanical Properties of Hydrogels

**Figure 6:**
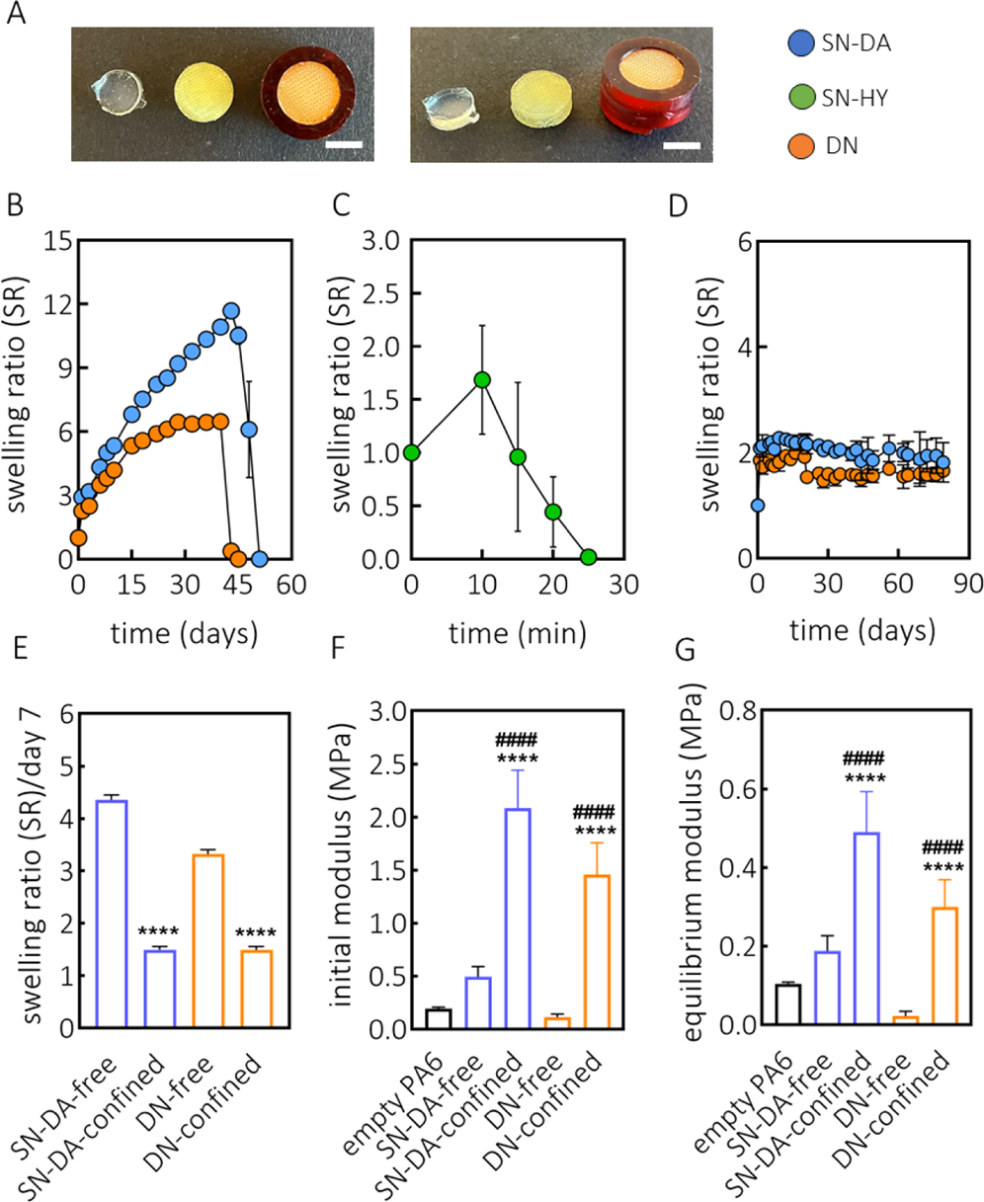
Swelling and mechanical properties. (A) Representative pictures of hydrogels prepared for free swelling and confined tests (example given by SN-DA). From left to right: free swelling hydrogel, semiconfined (formed within the spacer fabric scaffold, PA6, yellow color), and confined hydrogel (spacer fabric filled with hydrogel and inserted into a cassette). Swelling and mechanical tests were performed with free swelling and confined samples. Scale bar: 5 mm. (B) Swelling behavior of SN-DA (15 wt %, methylfuran-to-maleimide ratio of 5:1) and DN (15 wt %, DA-to-HY mass ratio of 1:1, methylfuran-to-maleimide ratio of 5:1, and aldehyde-to-hydrazone ratio of 1:1) under free swelling conditions (PBS, 37 °C). (C) Swelling profile of SN-HY (15 wt %, aldehyde-to-hydrazide molar ratio of 1:1) under free swelling conditions (PBS, 37 °C). (D) Swelling properties of SN-DA and DN (same composition as free swelling), under confined conditions (PBS, 37 °C), indicating superior stability and reduced swelling of both formulations. (E) Swelling ratio of SN-DA and DN hydrogel formulations, under free and confined swelling conditions, measured at day 7. (F) Initial modulus for SN-DA and DN (free swelling and confined) determined after compression of 15%. (G) Equilibrium modulus of free swelling and confined SN-DA and DN formulations following 15% compression. **** (####) *p* < 0.0001 (one-way ANOVA, Tukey’s multiple comparison test; * compared to the respective free hydrogel; # compared to the empty spacer fabric). Measurements were performed in triplicate (B–D) or sextuplicate (E–G).

After preparing different hydrogel formulations and characterizing them rheologically, we assessed their swelling behavior and mechanical properties. Mechanical performance of hydrogels is an important aspect to investigate as various biomedical applications require the material to be robust and stable. For applications in cartilage engineering, especially, mechanical stability under compression is relevant. Water-retention capacity and swelling behavior are related to changes in mechanical properties over time. Hydrogel discs were fabricated either as free or confined gels before testing (Figure 6A). Swelling capacity was calculated as the ratio between the weight of the swollen gel and its initial weight and was defined as the swelling ratio (SR).^34,58,59^Figure 6B shows the swelling ratio of SN-DA and DN hydrogels in time. Under free swelling in PBS and at 37 °C, both hydrogels steadily absorbed water in time, as observed from the increase of the corresponding swelling ratios. In detail, the SN-DA hydrogel exhibited a maximum SR of ∼12 at day 43, and it was fully degraded after 51 days. Degradation of SN-DA is related to the reversible character of the Diels–Alder adduct. The retro Diels–Alder reaction usually requires temperatures above 60 °C to occur, but it was reported taking place under physiological conditions as well, although very slowly.^60,61^ Moreover, there is another mechanism that could play a role in the degradation of the swelling hydrogel. The retro-Diels–Alder reaction is characterized by a relatively low activation energy (∼25 kcal/mol),^62^ making the DA adduct mechanically labile, when compared to other covalent bonds in polymer networks. Indeed, mechanically induced retro-Diels–Alder reactions have been reported before.^63−65^ In this case, we hypothesize that the physical expansion of the hydrogel sample was sufficient to put strain on the polymer chains (and cross-links), therefore driving the retro-Diels–Alder reaction. In either case, the reformed maleimide is susceptible to hydrolysis leading to ring opening. As this hydrolysis is nonreversible, it causes elimination of this functional group from the Diels–Alder/retro-Diels–Alder equilibrium and therefore permanent cleavage of the DA cross-links.^61^

Unlike SN-DA, DN swelled less extensively, reaching a maximum SR of ∼6.5 after 40 days (Figure 6B). The dissolution phase took until day 45, after which the gel was completely degraded. The shorter stability of the gel, compared to SN-DA, could be explained by the lower density of DA cross-links.

On the other hand, the SN-HY hydrogel displayed a very short stability in time (Figure 6C). In fact, the hydrogel reached a maximum SR of ∼1.7 after only 10 min and disintegrated completely within half an hour. This comes as no surprise, taking into account the dynamic character of the hydrazone bonds. These results are in agreement with the viscoelastic and stress relaxation data of HY cross-links reported in the previous sections.

We hypothesized that restraining hydrogel swelling could lead to osmotic pressure buildup and improvement in the material elastic modulus and stiffness. This osmotically induced stiffening could be useful for mimicking the properties of load-bearing tissues, such as cartilage. Therefore, we further investigated their swelling profile under confined swelling conditions. Hydrogels were prepared in a spacer fabric material (PA6) and inserted into a cassette, with the aim to physically restrain the hydrogel from swelling. Figure 6D shows the swelling profile for SN-DA and DN hydrogels when they were confined and immersed in PBS at 37 °C. After 10 days, the SR reached a maximum, ∼2.3 and 1.9 for SN-DA and DN, respectively, after which it was stable and equilibrated at ∼1.7 until the experiment was stopped (day 80). The swelling profile suggests that the physical confinement of the gel, and thus the impossibility to physically expand in response to water absorption and swelling, leads to improved stability in time. This observation indeed suggests that the predominant mechanism of the hydrogel swelling and degradation is likely the mechanically induced retro-Diels–Alder reaction of the DA adducts, as no significant physical expansion of the material could be achieved.

Related to the observed swelling behavior of these hydrogels, the assessment of their mechanical properties upon confinement was performed. Previously, it has been reported that swelling hydrogels under confined conditions led to osmotic pressure buildup and therefore osmotically induced stiffening of the construct.^66^ SN-DA and DN hydrogels were first prepared and allowed to swell for 7 days under both free swelling and confined conditions (Figure 6E). The SR of SN-DA was ∼4.3 for the free swelling sample, whereas for the confined one, it was found to be ∼1.5, with an achieved confinement of ∼65%. For DN, a total confinement of ∼55% was achieved, with SR changing from ∼3.3 for the free swelling sample to ∼1.5 for the confined sample. An ideal confinement would ensure a SR of 1; however, in this case, the final construct (cassette and the spacer fabric) had a partially open structure, therefore still allowing some swelling. These samples were subsequently used for mechanical testing by means of an indentation test up to 15% deformation, followed by a hold at constant deformation (15%) until an equilibrium was reached. From the quick step deformation, an initial modulus was derived, as shown in Figure 6F. Free swollen SN-DA displayed a modulus of ∼0.5 MPa, while the confined counterpart showed a modulus of ∼2 MPa (fourfold increase). Similarly, DN had a modulus of ∼0.1 MPa after 7 days under free swelling, which upon confinement reached values of ∼1.5 MPa. These results suggest that it was possible to induce pressurization in the system, leading to a significant increase in mechanical properties. However, considering that these materials are not purely elastic, but display viscoelastic properties (already discussed), it would be more accurate to discuss in terms of time-dependent behavior. The corresponding load relaxation profiles (at 15% deformation) were analyzed, and an equilibrium modulus was derived. As shown in Figure 6G, SN-DA possessed an equilibrium compressive modulus of ∼0.2 MPa when allowed to swell freely, which increased by a factor of 2.5 to ∼0.5 MPa under confined conditions. DN showed an increase from ∼0.02 to ∼0.3 MPa, changing from free to confined swelling. Such a remarkable increase in both initial and equilibrium moduli upon hydrogel confinement is in agreement with the previously reported swelling hydrogels.^66^ The overall contribution of the empty spacer fabric (confined) to the initial and equilibrium moduli was ∼0.2 and 0.1 MPa, respectively. For both moduli, the observed increase upon the confinement of hydrogels clearly originates from the restricted swelling. The difference in both moduli observed between SN-DA and DN hydrogels is most likely due to the inherent difference in swelling capacity between the free gels. The overall mechanical performance is determined by both the cross-linking density and restricted swelling-induced pressurization.

This feature is particularly interesting and useful in the development of cartilage-mimicking scaffolds. Cartilage tissue, due to its unique composition and structural organization with hydrated polyelectrolyte matrix and collagen fibers, is characterized by increased osmotic swelling pressure, which leads to the unique mechanical properties of cartilage. The confined hydrogel materials in this study are able to reproduce this aspect of cartilage tissue. The found values for the equilibrium compressive modulus of the present SN-DA and DN confined hydrogels are in proximity of the range expected for articular cartilage (0.36–1.11 MPa).^67^ These observations indicate that SN-DA hydrogels, especially, have potential as scaffolds for cartilage tissue engineering. Overall, these results indicate a new window of possibilities and potential applications, specifically related to cartilage tissue engineering.

### 3.7 Cytocompatibility of Hydrogels

**Figure 7:**
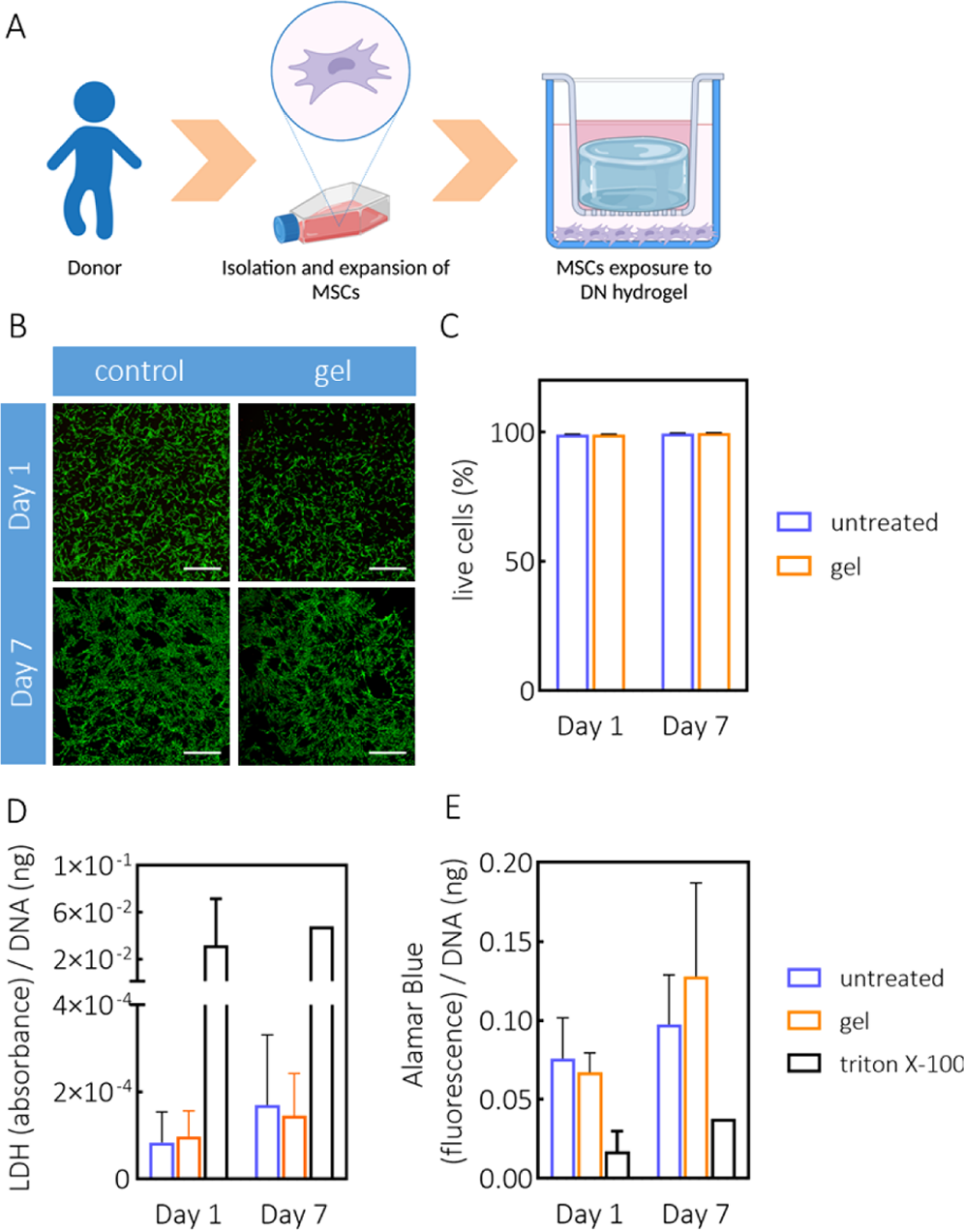
Cytocompatibility of DN hydrogel. (A) Experimental setup of MSC viability in the presence of DN hydrogel. Created with BioRender.com. (B) Representative images showing live cells (in green) upon treatment with DN hydrogel (15 wt %, DA-to-HY mass ratio of 1:1, methylfuran-to-maleimide ratio of 5:1, and aldehyde-to-hydrazone of 1:1), after 1 and 7 days. Scale bar: 1000 μm. (C) Quantification of live cells following Live/Dead assay, using ImageJ. (D) LDH release from cells measured after 1 and 7 days of treatment with DN gels and normalized against DNA content. Triton X-100 was used as a positive control. (E) Cell metabolic activity measured after 1 and 7 days of treatment with DN gels and normalized against DNA content. Triton X-100 was used as a negative control.

Following detailed material characterization, the next step was to assess the cytocompatibility of the hydrogel. MSCs were used for cell viability studies (live/dead, LDH release, and metabolic activity) in the presence of cross-linked DN hydrogels (15 wt %), using hanging cell culture inserts, and cytotoxicity was evaluated after 1 and 7 days (Figure 7A). Live/Dead images for 1 and 7 days are shown in Figure 7B, with majority of cells found to be alive when exposed to DN hydrogels. The percentage of live cells was not different between the treated and control conditions (Figure 7C). To confirm the high viability observed with the Live/Dead assay, additional tests were performed, aimed at assessing the release of LDH, as well as the metabolic activity of the cells when exposed to DN hydrogels for up to 7 days. In fact, gel-treated MSCs did not release an increased amount of LDH, a measure of cellular damage and cytotoxicity (Figure 7D). The metabolic activity also showed high cell viability, confirming the results obtained with the Live/Dead assay (Figure 7E). Concluding, the human MSCs exposed to fully cross-linked DN hydrogels were not affected but maintained high viability after 7 days.

We also investigated the effect of each individual component of the DN hydrogel, at concentrations relevant for fabricating DN hydrogels at 15 wt %, and it was found that after 1 h, none of the components caused cytotoxicity (Figure S7). Additionally, encapsulated ATDC5 cells were found to be viable after 24 h (Figure S8).

Overall, these preliminary cytocompatibility results indicate the suitability of the DN hydrogels for use in regenerative medicine applications. The high viability of MSCs after 7 days of exposure to DN gels supports future investigations for cell encapsulation studies and bioprinting applications.

### 3.8 DN Hydrogel Effects on Human Macrophages

**Figure 8:**
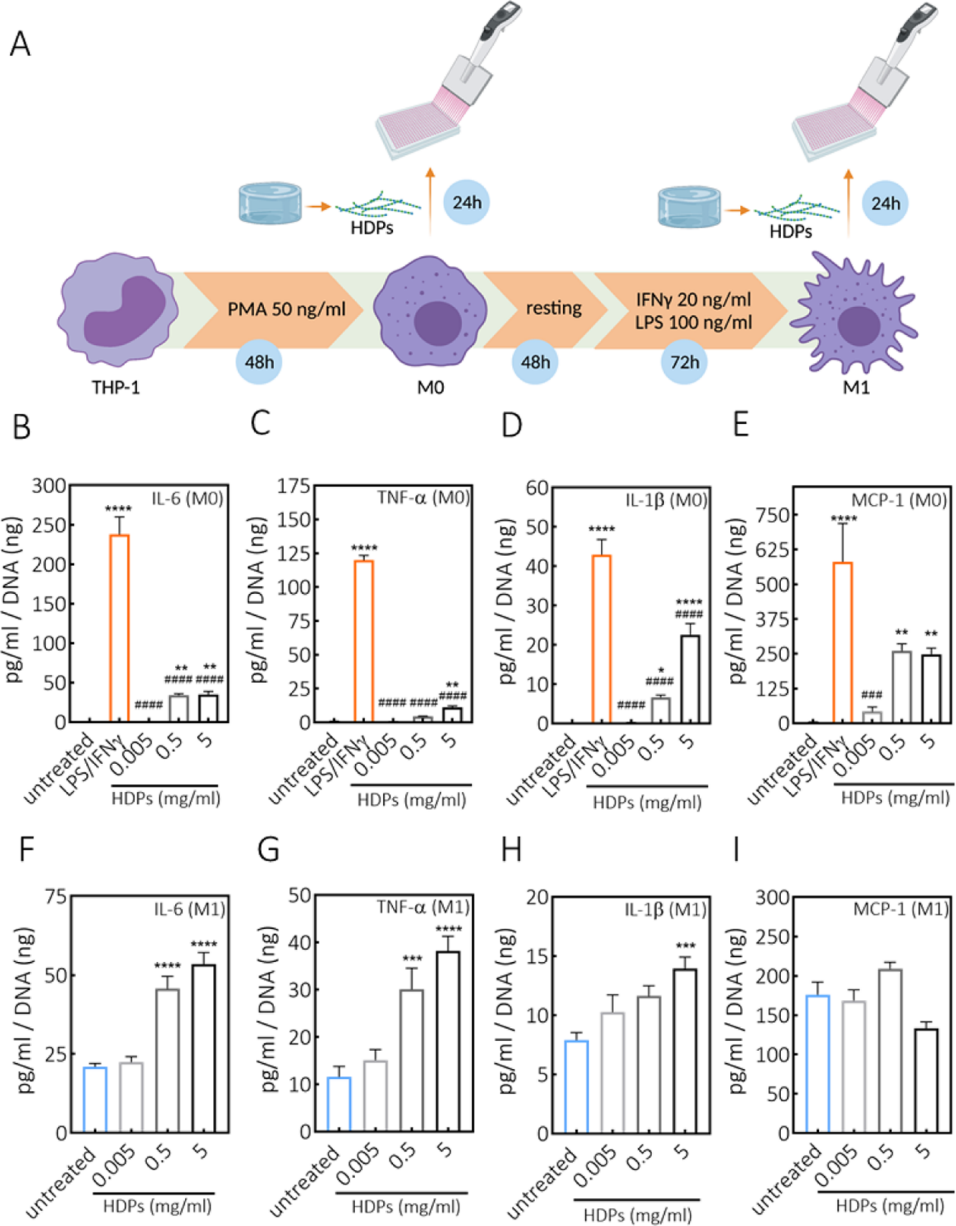
Effect of DN hydrogel degradation products on the cytokine production by THP-1-derived macrophages. (A) Schematic representation of human THP-1 differentiation to macrophages, polarization to M1 macrophages, and exposure to HDPs. Created with BioRender.com. (B–E) Release of IL-6, TNF-α, IL-1β, and MCP-1 cytokines by M0 macrophages. (F–I) Release of IL-6, TNF-α, IL-1β, and MCP-1 cytokines by M1 macrophages, as indicated in the figure plots, following 24 h treatments with increasing concentrations of HDPs, derived from DN hydrogel (15 wt %, DA-to-HY mass ratio of 1:1, methylfuran-to-maleimide of 5:1, and aldehyde-to-hydrazone of 1:1). *p < 0.05, **p < 0.01, ***p < 0.001, ****p < 0.0001, ###*p* < 0.001, ####*p* < 0.0001; * compared to untreated control, # compared to LPS/IFNγ treatment (one-way ANOVA, Tukey’s multiple comparison test). Measurements were performed in triplicate. PMA: phorbol 12-myristate 13-acetate.

Next, we studied the safety aspects regarding the potential inflammatory response to the biomaterials used in this study, in particular the effect of biomaterials on macrophages. These cells are key players in the host body reactions and, as such, are able to affect overall material’s biocompatibility.^68,69^ In relation to the potential application of DN gels for cartilage replacement therapies, as previously mentioned, it is even more important to check foreign body response features, as it is known that in inflammatory diseases of the joints macrophages play a crucial role in disease progression by releasing proinflammatory mediators.^70^ To study what the effect is of DN hydrogels on macrophages, DN hydrogel was incubated with HYAL II enzyme until complete material degradation. The recovered material, corresponding to the degraded hydrogel, is referred to as HDPs. HDPs mimic in vivo scenario, where the implanted hydrogel would be susceptible to enzymatic degradation. The THP-1 human monocyte cell line was used for differentiation toward M0 (nonactivated) and M1 (proinflammatory) macrophages, which were then exposed to HDPs. Both cell viability and release of osteoarthritis-related proinflammatory cytokines, including IL-6, TNF-α, IL-1β,^71^ and MCP-1,^72^ following exposure to HDPs were assessed. We assessed whether HDPs induce the activation of M0 toward M1 macrophages, as well as exacerbate the activity of M1. The experimental setup is schematically depicted in Figure 8A. Exposure of M0 and M1 macrophages to increasing concentrations of HDPs did not lead to cytotoxicity when compared to positive control (Figure S9), indicating that HDPs are not cytotoxic, which is in line with the observed results with MSCs and DN hydrogel. Figure 8B–E shows the cytokine release by M0 macrophages upon exposure to HDPs. The secreted levels of all investigated cytokines showed a dose-dependent increase. However, HDPs do not seem to be as efficient as LPS and IFNγ to activate M0 macrophages, which were used as controls to mimic the maximum stimulation of M0 and polarization toward the M1 phenotype. Next, the effect of HDPs was also evaluated on the activated M1 macrophages (after having removed the medium from LPS/IFNγ treatment). Figure 8F–I shows the cytokine release profiles when M1 macrophages were exposed to HDPs. Also in this case, the lowest tested concentrations did not significantly influence cytokine production by M1 macrophages, thus not exacerbating their activity and proinflammatory capacity. At higher concentrations, in particular 5 mg/mL, the levels of IL-6, TNF-α, and IL-1β were significantly increased, whereas the MCP-1 production by M1 was not affected at any given concentration of HDPs.

It has been reported that HA at low molecular weights (<64 kDa^73,74^) is considered a DAMP (damage-associated molecular pattern) stimulus, mediating inflammatory reactions.^75,76^ HDPs used in this study contain fragments of HA, at molecular weights lower than 80 kDa. Therefore, it can be expected that at increasing HDP concentrations, the amount of HA also increases, thus leading to inflammatory events. On the other hand, CS, which is also present in HDPs, has been reported to have anti-inflammatory properties, by reducing the production of proinflammatory cytokines TNFα and IL-1β.^77,78^ In contrast, CS has also been shown to exhibit proinflammatory properties in central nervous system disorders, through integration of signals from the microenvironment and activation of immune cells.^79^ In our study, however, HDPs are composed of a mixture of CS and HA at low molecular weights, and therefore the overall response might depend on the balance between the two components and their biological activity.

In view of the possible regenerative cartilage applications, the concentrations of HDPs used in this experiment were in the range of the concentrations that would be reached if the DN hydrogel (diameter 1 cm, height 2 mm, 15 wt %) was fully and instantly degraded within the synovium of the knee joint (∼3.5 mL), without taking the clearance of the synovial fluid into consideration. Therefore, it is highly unlikely that the highest investigated concentrations will be reached in an in vivo setting, thus arguing for a safe application of herein developed DN hydrogel. However, the safety aspects related to a possible clinical translation remain to be further investigated in appropriate animal models, from which more comprehensive and physiologically relevant conclusions could be drawn. In this study, sterilization of the materials (ethanol for the spacer fabric and filtration for the polymers) was done for in vitro studies, but for translational applications, sterilization of the final implant should be taken into consideration.

## 4 Conclusions

In this study, we investigated the suitability of the combination of dynamic bonds with different lifetimes, to produce CS/HA-based DN hydrogels that exhibit a wide range of useful properties, especially tunable viscoelasticity and stress relaxation, while maintaining stability and integrity. The integration of Diels–Alder and hydrazone cross-links in a single formulation proved to be an efficient way to impart hydrogel processability and self-healing features initially (due to quickly formed dynamic hydrazone bonds) but without sacrificing the long-term structural integrity (due to Diels–Alder adducts). Importantly, the DN hydrogel could be easily fabricated in situ (pH 7.4, 37 °C), without applying external stimuli (e.g., UV light, temperature, or changes in pH). Rheological analysis showed that hydrazone cross-links displayed short lifetimes, in the order of ∼800 s, whereas Diels–Alder cross-links proved to be rather stable at experimental timescales. Moreover, the stress relaxation profile and viscoelasticity could be easily tuned by changing the mass ratio of Diels–Alder and hydrazone components. The results also demonstrated that DN hydrogel has the possibility to be used in extrusion-based 3D printing. Furthermore, both DN and SN-DA hydrogels showed remarkable swelling capacity and stability. The bulk degradation under physiological conditions can be predominantly ascribed to the mechanically induced retro-Diels–Alder reactions. However, by physically restraining the hydrogels, stability was significantly improved, in addition to the osmotically induced pressurization leading to stiffened constructs, which could potentially mimic cartilage mechanical properties. Finally, the prepared hydrogels were noncytotoxic to MSCs over 7 days, further demonstrating the attractive properties of the present biopolymer-based gels for biomedical applications. In the following studies, this system will be further explored as a bioink. Additionally, future studies will be directed toward cartilage regeneration applications, taking into consideration the promising swelling and mechanical properties found here. When moving toward translational applications, sterilization aspects should be taken into consideration.

The approach employed in this study could be easily translated to other types of cross-linking chemistries (covalent, physical, or dynamic covalent bonds) in order to achieve tunable viscoelasticity of hydrogel materials without sacrificing mechanical integrity and stability. We envision that multicomponent hydrogels (e.g., DN hydrogels) with different combinations of dynamic cross-links could lead to smart, responsive, and multifunctional soft materials with tunable and customizable properties.^25^

## Supporting Information Available

^1^H NMR spectra of HA-MeFU and CS-mal; FT-IR spectra of HA-MeFU, CS-mal, and CS-ox; strain sweep measurements of SN-DA and SN-HY; dynamic amplitude sweep of SN-HY; gelation of SN-HY; strain sweep of DN hydrogel; gelation of DN hydrogel with initial shearing; Live/Dead quantification of the MSCs treated with hydrogel components; Live/Dead analysis of encapsulated ATDC5 cells and related methods; and viability of M0 and M1 macrophages upon treatment with hydrogel degradation products (PDF)
