# Supplementary material for: Viscoelastic Chondroitin Sulfate and Hyaluronic Acid Double-Network Hydrogels with Reversible Cross-Links
Source: Biomacromolecules. 2022 Feb 23;23(3):1350–65. doi: 10.1021/acs.biomac.1c01583 (PMC8924925; doi:10.1021/acs.biomac.1c01583)
Supplement: Supplementary file 1 — bm1c01583_si_001.pdf [file bm1c01583_si_001.pdf]

SUPPORTING INFORMATION

Viscoelastic Chondroitin Sulfate and Hyaluronic Acid Double-Network Hydrogels with Reversible Crosslinks

Marko Mihajlovic[1,2], Margot Rikkers[3‡], Milos Mihajlovic[4,‡], Martina Viola[1,3], Gerke Schuiringa[2], Blessing C. Ilochonwu[1], Rosalinde Masereeuw[4], Lucienne Vonk[3], Jos Malda[3,5], Keita Ito[2,3] and Tina Vermonden[1]*

[1]*Department of Pharmaceutics, Utrecht Institute for Pharmaceutical Sciences (UIPS), Utrecht University, Utrecht, the Netherlands*
[2]*Department of Biomedical Engineering, Eindhoven University of Technology, Eindhoven, the Netherlands*
[3]*Department of Orthopaedics, University Medical Center Utrecht, Utrecht University, Utrecht, The Netherlands*
[4]*Department of Pharmacology, Utrecht Institute for Pharmaceutical Sciences (UIPS), Utrecht University, Utrecht, the Netherlands*
[5]*Department of Equine Sciences, Faculty of Veterinary Medicine, Utrecht University, Utrecht, Netherlands*

‡ These authors contributed equally
* Corresponding author: Prof. Dr. Tina Vermonden, T.Vermonden@uu.nl

Table S1. Summary of compositions for hydrogel formulations used in the present work.

materials	maleimide:methylfuran molar ratio	aldehyde:hydrazide molar ratio	ADH crosslinker	mass ratio DA:HY	total polymer
SN-DA	1:1	n/a	no	1:0	15 wt%
	2:1	n/a	no	1:0	15 wt%
	5:1	n/a	no	1:0	15 wt%
SN-HY	n/a	1:0.75	yes	0:1	15 wt%
	n/a	1:1	yes	0:1	15 wt%
	n/a	1:1.25	yes	0:1	15 wt%
	n/a	1:1	yes	0:1	10 wt%
DN	5:1	1:1	yes	1:1	15 wt%
	5:1	1:1	yes	2:1	15 wt%

n/a not applicable

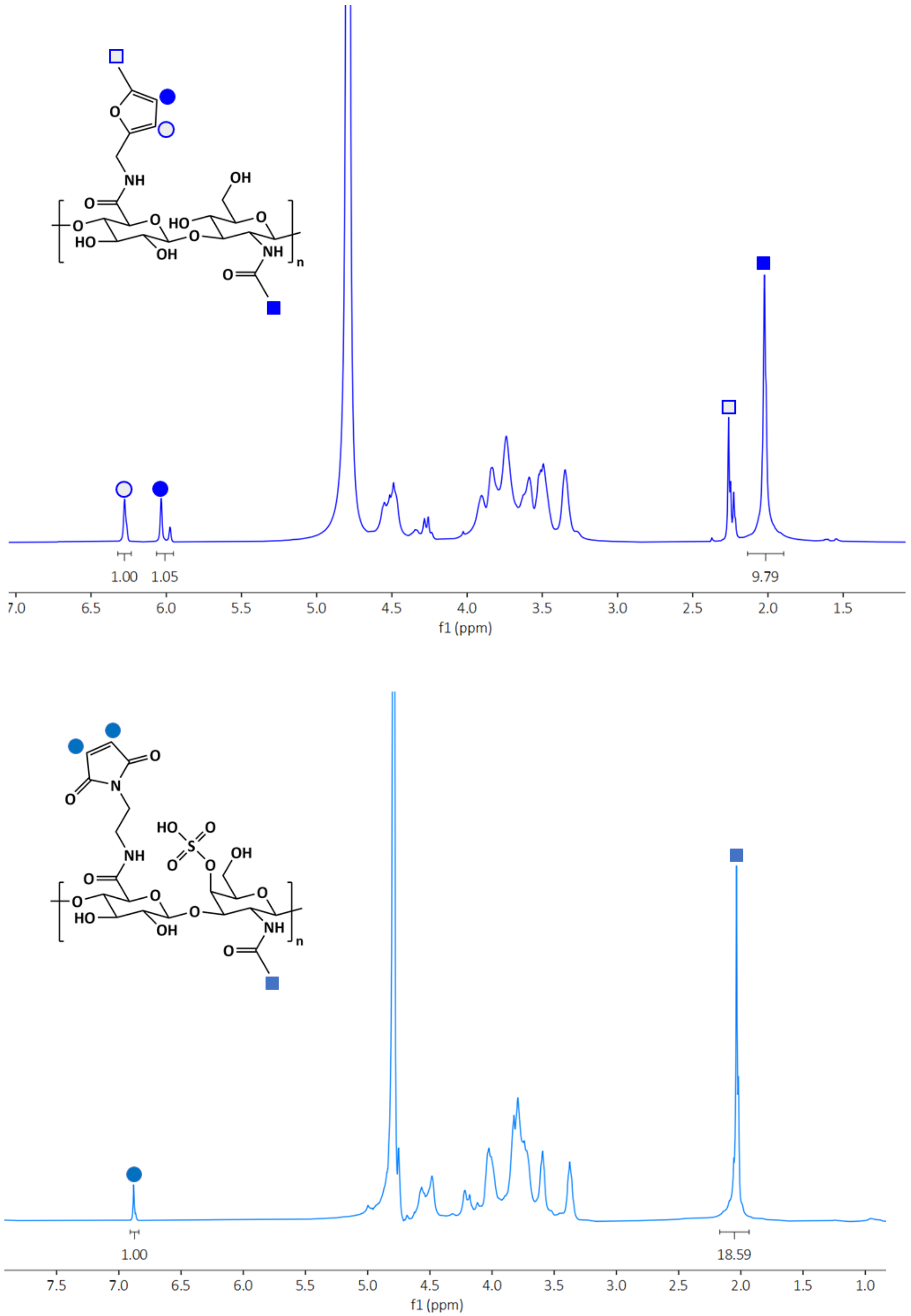

Figure S1. ^{1}H-NMR characterization of HA-MeFU (top) and CS-mal (bottom). Modification of HA with 5-methylfuran was quantified by comparison of the integrals of the *N*-acetyl glucosamine peak (3 H, 2.0 ppm) with

the aromatic methylfuran peaks (1 H, 6.0 ppm and 1 H, 6.3 ppm), resulting in modification of ~30% of HA repeating units. Degree of substitution of CS-mal was determined by comparison of the integrals of the maleimide peak (2 H, 6.9 ppm) to the methyl peak of the polysaccharide backbone (3 H, 2.0 ppm) and determined to be ~8% of disaccharide repeats of CS.

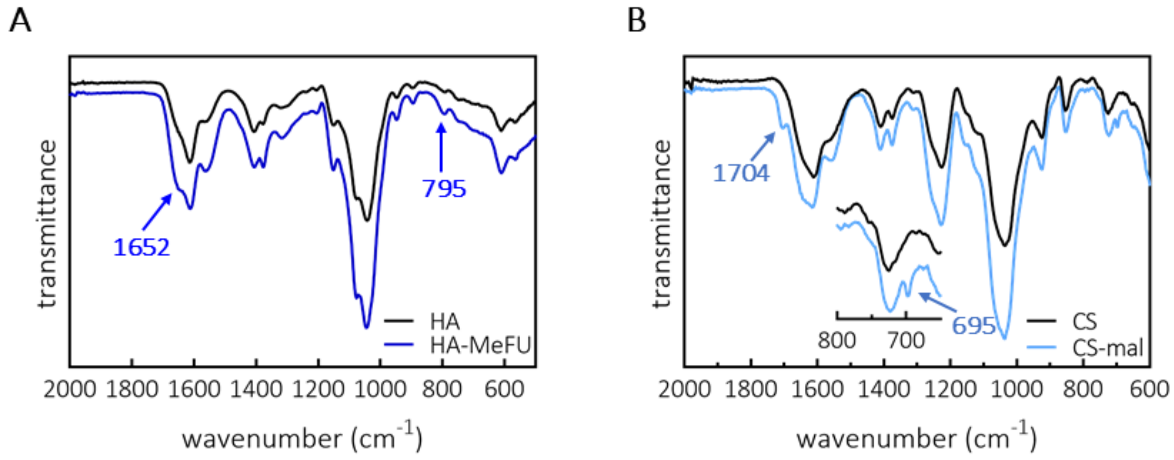

Figure S2. FT-IR characterization of HA-MeFU and CS-mal. A) FT-IR spectra of HA (black) and HA-MeFU (blue) polymers, with arrows indicating -C=O- stretch typical of amide formation (v = 1652 cm^{-1}) and =C-H- bend of furan moiety (v = 795 cm^{-1}). B) FT-IR spectra of CS (black) and CS-mal (blue) polymers. Arrows indicate -C=O stretch of maleimide (v = 1704 cm^{-1}) and =C-H- bend of maleimide (v = 695 cm^{-1}).

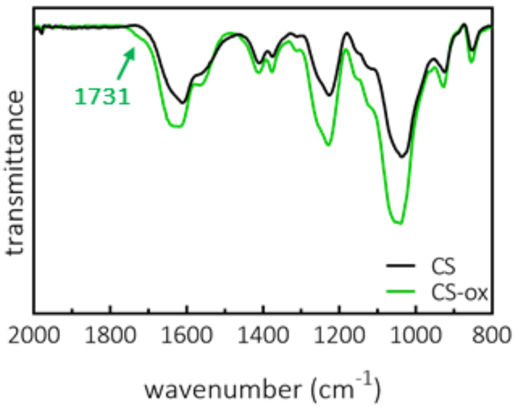

Figure S3. FT-IR characterization of CS-ox. FT-IR spectra of CS (black) and CS-ox (green) polymers. Arrow indicates the characteristic -C=O- stretching vibration of the aldehyde functionalities (v = 1731 cm^{-1}).

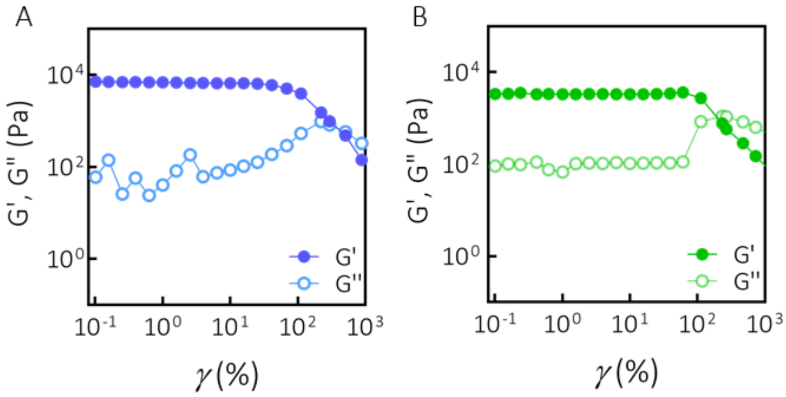

Figure S4. Linear viscoelastic regime of single-network hydrogels. A) Strain sweep test (ω = 1 rad/s, γ = 0.1-1000%) of SN-DA at 15 wt% (methylfuran to maleimide ratio of 5:1). B) Strain sweep test (ω = 1 rad/s, γ = 0.1-1000%) of SN-HY at 15 wt% (aldehyde to hydrazide ratio of 1:1).

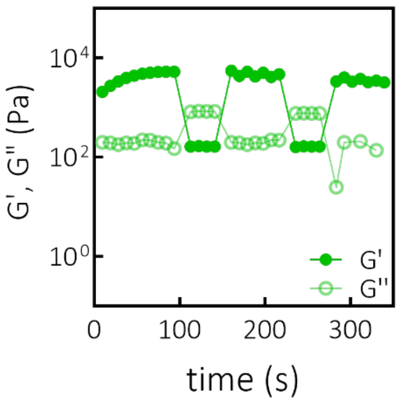

Figure S5. Reversibility of the hydrazone crosslinks. Dynamic amplitude test of SN-HY (15 wt%, aldehyde to hydrazide ratio 1:1). Hydrogel was subjected to time sweep tests at low (ω = 1 rad/s, γ = 1%, first cycle 120s, following cycles 60s) and high oscillation strains (ω = 1 rad/s, γ = 500%, 45s).

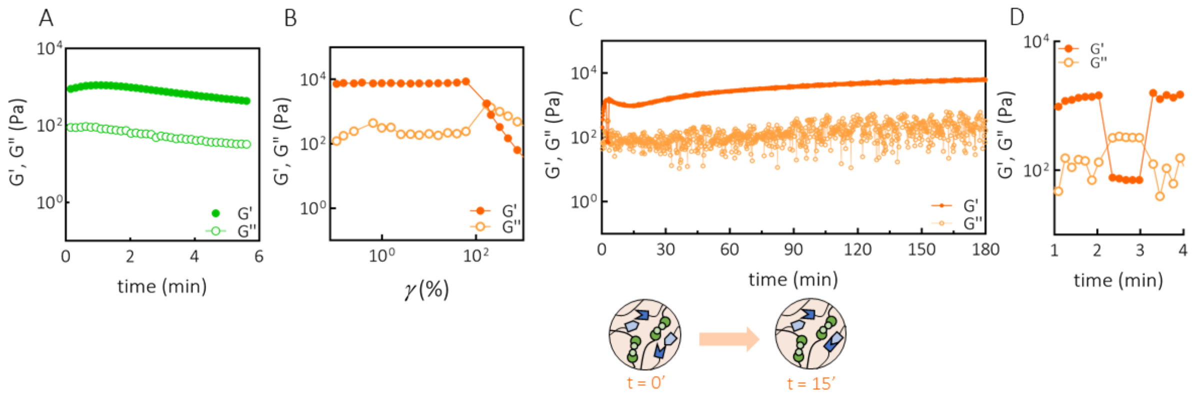

Figure S6. Linear viscoelastic region and gelation with initial shearing of double-network hydrogel. A) Time sweep (ω = 1 rad/s, γ = 1%) of SN-HY hydrogel (7.5 wt%, aldehyde to hydrazide 1:1) at 37 ^{0}C. B) Strain sweep test (ω = 1 rad/s, γ = 0.1-1000%) of DN hydrogel (15 wt%, SN-DA to SN-HY 1:1, methylfuran to maleimide 5:1, aldehyde to hydrazide 1:1). C) Gelation (ω = 1 rad/s, γ = 1%) of DN at 15 wt% (SN-DA to SN-HY mass ratio of 1:1, methylfuran to maleimide molar ratio of 5:1, aldehyde to hydrazide molar ratio of 1:1) with initial shearing applied. D) Zoomed in portion of the initial shearing (ω = 1 rad/s, γ = 500%, 45 s) and immediate recovery of the starting stiffness.

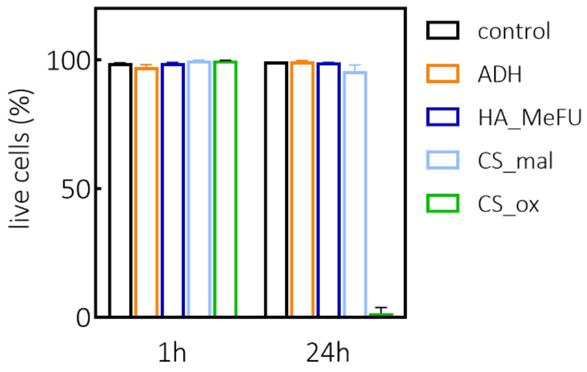

Figure S7. Cytocompatibility of the individual hydrogel components. A) Live/Dead results of MSCs treated with the polymers and crosslinker after 1 and 24h. Following concentrations were used: ADH (1.4 mg/mL), HA-MeFU (37.5 mg/mL), CS-mal (37.5 mg/mL) and CS-ox (75 mg/mL), the concentrations necessary to fabricate 15 wt% DN hydrogel (DA to HY mass ratio 1:1).

The cell monolayer was treated with each component for 24h and the percentage of live cells was quantified. Importantly, after one hour, none of the components induced a decrease in cell viability. However, after 24h of exposure, CS-ox polymer caused a severe cytotoxic effect. The observed toxic effect of CS-ox was likely due to the presence of highly reactive aldehyde functionalities, which react promptly with nucleophilic groups, such as primary amino groups, abundantly present in biological media containing cells [1]. However, for cell encapsulation studies, the observed toxicity of CS-ox is not expected to be of major concern, as the gel components will not be freely exposed to cells for long times and we showed that cytotoxicity was not observed after 1h exposure. In particular, CS-ox was shown to form hydrazone bonds instantly in the presence of the ADH crosslinker, therefore the time at which aldehyde groups are directly exposed to cells will be minimized in the presence of ADH. Additionally, in a recent work, studying oxidized alginate-based hydrogels and making use of hydrazone crosslinks, it was shown that upon cell encapsulation within 2% (w/v) hydrogel, the cell viability was not affected, suggesting that the aldehyde groups were engaged in the network formation, rather than reacting with cell components [2].

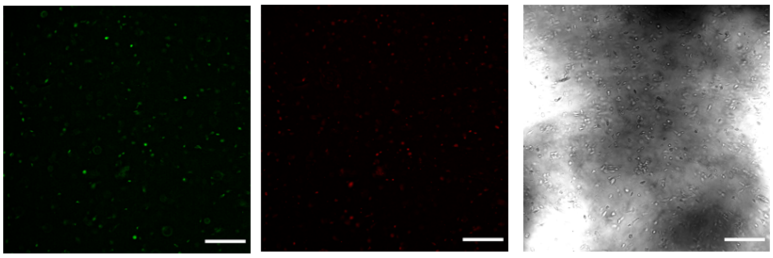

Figure S8. Cytocompatibility of encapsulated cells after 24 h. Representative Live/Dead and brightfield images of encapsulated ATDC5 cells within DN hydrogel (15 wt%, DA to HY mass ratio 1:1), scale bar 200 µm.

Cell encapsulation in hydrogel formulation

ATDC5 cells were resuspended at 7.5x10^6/mL in DN gel precursor (15 wt%) and cylindrical gels were prepared as described before (Teflon mold, 2 x 6 mm, incubated for 45 minutes at 37°C). Upon incubation, cell-laden gels were transferred to a culture plate with high glucose Dulbecco's modified Eagle's medium (DMEM; Sigma-Aldrich) supplemented with 1% (v/v) penicillin/streptomycin (pen/strep; 100 U/mL, 100 µg/mL; Gibco), 2% (v/v) human serum albumin (HSA; Albuman,

Sanquin Blood Supply Foundation), 1% insulin-transferrin-selenium-ethanol-amine (ITS-X; Gibco), and 200 µM l-ascorbic acid 2-phosphate (ASAP; Sigma-Aldrich).

Cell viability following hydrogel encapsulation

DN gels were analyzed after one day in culture for viability and cell distribution using the Live/Dead Viability/Cytotoxicity Kit for mammalian cells according to the manufacturer's instructions (Thermo Fisher). Images were acquired on a THUNDER fluorescence microscope and LASX acquisition software (both Leica microsystems, Wetzlar, Germany).

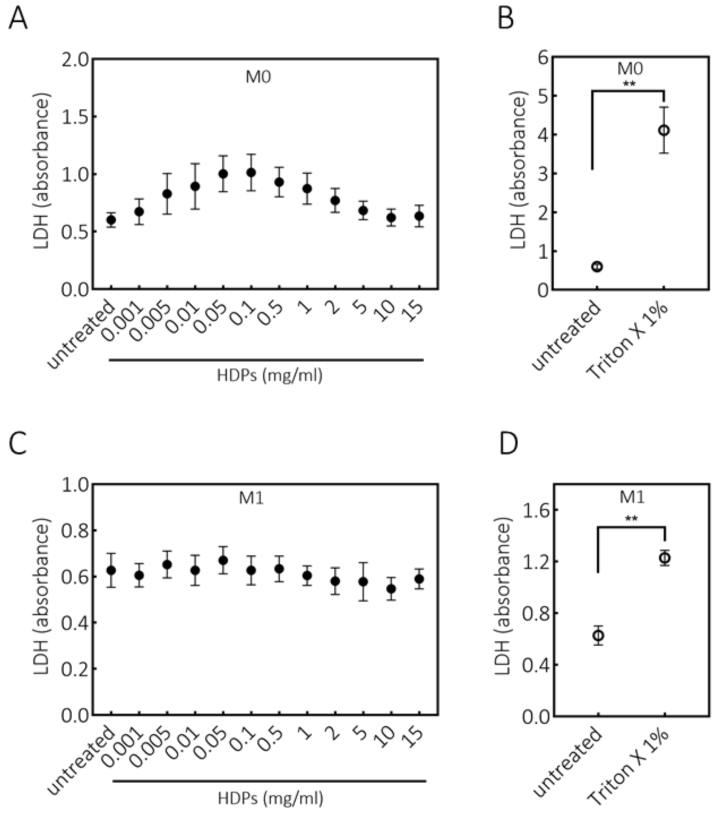

Figure S9. HDPs effect on macrophage viability. A) LDH release by M0 macrophages upon 24h exposure to increasing concentrations of HDPs. B) LDH release from M0 macrophages treated with Triton X-100 1% (positive control). C) LDH release by M1 macrophages upon 24h exposure to increasing concentrations of HDPs. D) LDH release from M1 macrophages treated with Triton X-100 1% (positive control). ** $p < 0.01$ (unpaired t test). Measurements were performed in triplicate.

REFERENCES

1. LoPachin, R. M.; Gavin, T., Molecular mechanisms of aldehyde toxicity: a chemical perspective. *Chem Res Toxicol* **2014,** *27* (7), 1081-91.
2. Hafeez, S.; Ooi, H. W.; Morgan, F. L. C.; Mota, C.; Dettin, M.; Van Blitterswijk, C.; Moroni, L.; Baker, M. B., Viscoelastic Oxidized Alginates with Reversible Imine Type Crosslinks: Self-Healing, Injectable, and Bioprintable Hydrogels. *Gels* **2018,** *4* (4) 85.
